# Striatal cholinergic interneuron membrane voltage tracks locomotor rhythms in mice

**DOI:** 10.1038/s41467-023-39497-z

**Published:** 2023-06-26

**Authors:** Sanaya N. Shroff, Eric Lowet, Sudiksha Sridhar, Howard J. Gritton, Mohammed Abumuaileq, Hua-An Tseng, Cyrus Cheung, Samuel L. Zhou, Krishnakanth Kondabolu, Xue Han

**Affiliations:** 1 Department of Biomedical Engineering, Boston University, Boston, MA USA; 2 Department of Comparative Biosciences, University of Illinois at Urbana-Champaign, Urbana, IL USA

**Keywords:** Basal ganglia, Membrane potential, Neural circuits

## Abstract

Rhythmic neural network activity has been broadly linked to behavior. However, it is unclear how membrane potentials of individual neurons track behavioral rhythms, even though many neurons exhibit pace-making properties in isolated brain circuits. To examine whether single-cell voltage rhythmicity is coupled to behavioral rhythms, we focused on delta-frequencies (1–4 Hz) that are known to occur at both the neural network and behavioral levels. We performed membrane voltage imaging of individual striatal neurons simultaneously with network-level local field potential recordings in mice during voluntary movement. We report sustained delta oscillations in the membrane potentials of many striatal neurons, particularly cholinergic interneurons, which organize spikes and network oscillations at beta-frequencies (20–40 Hz) associated with locomotion. Furthermore, the delta-frequency patterned cellular dynamics are coupled to animals’ stepping cycles. Thus, delta-rhythmic cellular dynamics in cholinergic interneurons, known for their autonomous pace-making capabilities, play an important role in regulating network rhythmicity and movement patterning.

## Abstract (web summary)

Behaviorally relevant neural rhythms have been mainly studied at the neural population level. Here, the authors show that subthreshold membrane voltage delta-frequency oscillations in individual striatal cholinergic neurons modulate spike timing, striatal network beta rhythmicity, and track patterned stepping movement.

## Introduction

Rhythmic neural activities are broadly observed across neural circuits and linked to behavior. Thus far, behaviorally relevant neural rhythms have been mainly studied by measuring electrical field potentials at the population circuit level. One particular rhythm, the delta (1–4 Hz) rhythm, is prominent not only at the circuit level, but also at the behavioral level^1–6^. Neural circuit delta frequency oscillations occur across various motor circuits and are thought to provide a network coordination mechanism during locomotion^4–6^. Interestingly, stepping movements also occur at delta frequencies in vertebrates including humans and mice^1–4^. Locomotion depends on the central pattern generators in the spinal cord that exhibit autonomous pacemaking activity to orchestrate muscle movements, which are extensively modulated by supra-spinal descending inputs^7–9^. The frequency similarity between neuronal delta rhythmicity and stepping cycles suggests that supra-spinal motor circuit activity may regulate movement patterning via frequency-dependent circuit coupling. Indeed, neurons in the motor cortex and the cerebellum exhibit stepping-related spiking activity at delta frequencies^10–14^ and sensory stimulation at delta frequencies improves gait and mobility in Parkinsonian patients^15–18^. However, it is unknown whether similar movement-related delta-rhythmic temporal patterns occur in the striatum, the major input nucleus of the basal ganglia and a key structure for coordinated motor control that reciprocally interacts with the motor cortex and other subcortical motor circuits.

Delta rhythmic neural activity, typically measured as local field potential (LFP) delta oscillations, has been shown to organize higher frequency LFP oscillations at beta (~20–40 Hz) and gamma (>35 Hz) frequencies across the cortico-basal ganglia-thalamic circuit^4,19–21^. While prominent in layered cortical structures, LFP oscillations in the striatum are generally weak due to the lack of neuronal electrical dipole configurations. Nonetheless, transient fluctuations in striatal LFP beta oscillations are related to discrete aspects of motor behavior and are coordinated by cortical and thalamic delta oscillations^4,19,22–26^. Exaggerated beta oscillations in particular are considered a functional biomarker for Parkinson’s disease^4,19,20^.

So far, examination of the subthreshold membrane voltage (Vm) of individual striatal neurons in the brain has been limited to anesthetized animals^26–29^ because of the difficulty of performing intracellular recordings during behavior. It, therefore, remains unclear how the Vm of different striatal neuron subtypes with distinct biophysical properties are dynamically modulated during behavior. Among various striatal neuron subtypes, cholinergic interneurons (ChIs) likely play a prominent role in regulating striatal network dynamics during movement due to their unique ability to influence the striatal network through extensive anatomical arborizations^30–32^. One prominent biophysical feature of ChIs is their autonomous pacemaking behavior and subthreshold membrane voltage resonance at delta frequencies as reported in many patch clamp studies in brain slices^33,34^ and in anesthetized animals^26–29^. Further, ChIs exhibit movement-related activities^35,36^ and are also broadly implicated in Parkinson’s disease, particularly in the pathophysiology of falls and the freezing of gait^30,31,37^, and anti-cholinergic drugs are effective in managing certain Parkinsonian motor symptoms^37^. Despite the important role of ChIs in regulating striatal circuit activity in both normal and pathological conditions^20,35,36,38–40^, it remains largely unclear how the cellular dynamics of ChIs relate to striatal circuit dynamics during locomotion.

To probe how cellular voltage dynamics of individual neurons, particularly ChIs in the dorsal striatum, contribute to rhythmic locomotion, we performed simultaneous voltage imaging of individual neurons’ Vm and spiking with LFP recordings in mice during voluntary movement. We imaged membrane voltage at the soma of ChIs expressing the genetically encoded, soma-targeted voltage indicator SomArchon^41^, and compared ChI responses to those of non-specific striatal neurons, dominated by striatal spiny projection neurons (SPNs).

## Results

### Optical voltage imaging reveals that most cholinergic interneurons (ChIs), and a subset of non-specific striatal neurons (SYNs), exhibit spike bursting at delta frequencies (1–4 Hz) during locomotion

**Fig. 1:**
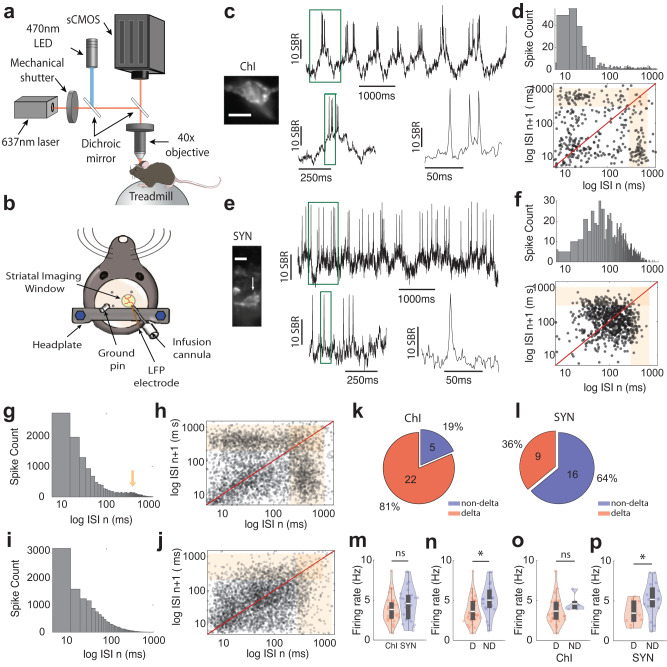
Membrane voltage imaging of individual striatal neurons (SYNs) and cholinergic interneurons (ChIs) reveals prominent cellular voltage delta rhythmicity in mice during locomotion. **a**, **b** Illustration of the (**a**) experimental setup and (**b**) surgically-placed imaging window. **c** An example ChI’s SomArchon fluorescence trace recorded at 833 Hz (left bottom, mean SomArchon fluorescence of the recorded neuron). Right, an example SomArchon fluorescence trace, with a zoom-in view (below) of the period indicated by the green box, and a further zoom-in (right). **d** Top, the inter-spike interval (ISI) distribution of the example ChI shown in **c**. Bottom, the ISI return map. The delta frequency range is illustrated with orange shading. **e** Same as **c**, but for an example SYN. **f** Same as **d**, but for the example SYN shown in **e**. Scale bars are 15 μm. **g** The population ISI distribution of delta-rhythmic neurons (*n* = 31). Arrow indicates the delta time-scale ISI distribution peak. **h** The ISI return map of all delta-rhythmic neurons (*n* = 31). **i**, **j** The population ISI distribution (**i**) and ISI return map (**j**) of non-delta neurons (*n* = 21). **k**, **l** Pie chart illustration of the fraction of (**k**) ChI neurons and (**l**) SYN neurons identified as delta-rhythmic or non-delta rhythmic. **m** Population firing rate for ChIs and SYNs. There was no difference between ChIs and SYNs (independent *t*-test, *p* = 0.16, *n* = 52, with 27 ChIs and 25 SYNs, df = 50). **n** Population firing rate for delta-rhythmic (D) and non-delta (ND) neurons (independent *t*-test, *p* = 0.007, *n* = 52, with 31 delta-rhythmic neurons and 21 non-delta neurons, df = 50). **o** Mean firing rate quantification for delta-rhythmic and non-delta ChIs (independent *t*-test, *p* = 0.26, *n* = 27, df = 25). **p** Same as **o**, but for SYNs (independent *t*-test, *p* = 0.04, *n* = 25, df = 23). Quantifications in **m**–**p** are visualized as violin plots with the outer shape representing the data kernel density and a box-and-whisker plot (box: interquartile range, whiskers: 1.5x interquartile range, white line: mean). All statistical tests are two-sided. Source data are provided as a Source Data file.

To measure membrane voltage from individual striatal neurons along with local field potentials (LFPs) during locomotion, we surgically implanted custom imaging windows coupled with an infusion cannula and an LFP electrode over the dorsal striatum (Fig. 1a, b). AAV viral vectors were then infused through the infusion cannula to transduce individual striatal neurons with the genetically encoded, soma-targeted voltage indicator SomArchon^41^ (Fig. 1c, e left). SomArchon voltage imaging of cholinergic interneurons (ChIs) was performed by either infusing Cre-dependent AAV-FLEX-SomArchon-GFP into ChAT-Cre mice or using tdTomato (tdT) fluorescence to identify ChIs in ChAT-tdT mice infused with AAV-syn-SomArchon-GFP (*n* = 6 mice). To compare ChIs to the general striatal neuron population, we infused AAV-syn-SomArchon-GFP into the striatum to transduce neurons expressing synapsin (SYNs), a non-specific neuronal marker (*n* = 7 mice). Histological quantification confirmed that 84.5 ± 7.2% (mean ± standard deviation, *n* = 34 fields of view in 17 brain slices from four mice, containing a total of 836 neurons) of transduced SYNs were SPNs, expressing the SPN-specific protein DARPP-32 (Supplementary Fig. 1) similar to previous observations^42^.

Striatal LFP recordings and ChI and SYN single-cell membrane voltage traces were obtained as head-fixed mice ran voluntarily on a treadmill made of a ball freely rotating along its center axis (Fig. 1a). Due to the need for high imaging speeds to capture the millisecond time-scale membrane voltage fluctuations in individual neurons, voltage imaging was performed with a custom widefield microscope at a frame rate of 833 Hz (Fig. 1a). During each recording, we first identified SomArchon-expressing cells based on the fluorescence of soma-targeted GFP (fused to SomArchon) under a 10X objective. We then collected near-infrared SomArchon fluorescence from the identified neurons under a 40X objective lens to maximize photon collection efficiency. We restricted the illumination of the near-infrared 637 nm laser to a circular area of ~70 µm in diameter to minimize background SomArchon fluorescence excitation under the widefield microscopy configuration, resulting in a field of view (FOV) size of about 50 µm × 70 µm.

During offline data analysis, we first performed motion correction of the recorded video to correct fine image motion, inherent to imaging studies in awake behaving animals (details in Methods). Since SomArchon expression is restricted to the soma and proximal dendrites, with little detectable expression in dendrites beyond 30 µm from the soma^41^, we manually segmented the cell body based on SomArchon fluorescence and extracted SomArchon fluorescence traces. SomArchon fluorescence traces were visually inspected to ensure minimal image motion and reasonable signal-to-noise level based on spike appearance. Trials with low spike signal-to-noise were removed and time periods with image motion exceeding 0.065 µm/s were excluded from further analysis. We then identified spikes from SomArchon membrane voltage traces and subsequently calculated the subthreshold membrane voltage trace (Vm) at the soma by removing the identified spikes.

ChIs are known to be tonically active^32^, and we indeed detected persistent spiking across all recorded ChIs, with many exhibiting delta-rhythmic (1–4 Hz) spike bursting accompanied by large Vm depolarizations (Fig. 1c). To quantify spike rhythmicity, we calculated the inter-spike interval (ISI) distribution across all spikes for each recorded neuron (representative ChI and SYN in Fig. 1d, f, respectively). In most ChIs and a small subset of SYNs, we detected two ISI peaks: one centered around 10–40 ms corresponding to high-frequency spike bursting at 25–100 Hz, and the other at approximately 300–700 ms (1.4–3.3 Hz) largely within the delta frequency range (orange-shaded region, Fig. 1d and Supplementary Fig. 2a, b). The remaining majority of SYNs and the few remaining ChIs, however, exhibited more regularly spaced spiking patterns (Fig. 1e, f and Supplementary Fig. 2c, d).

Given the prominence of delta-rhythmic spike bursting observed in many recorded neurons, we categorized each ChI and SYN as either delta-rhythmic (“delta”) or non-delta-rhythmic (“non-delta”) based on the ratio of the fraction of ISIs at 300–700 ms to the fraction of ISIs at 80–200 ms (Supplementary Fig. 3), a measure that captures the delta rhythmicity. The classified neurons’ ISI return maps, which plot the inter-spike interval relative to the subsequent spike (ISI n vs. ISI n + 1), were also manually inspected to confirm delta rhythmicity. Across the delta neuron population, the ISI distribution map had a prominent peak around delta frequency (2–3 Hz) (Fig. 1g, h), while non-delta neurons did not exhibit a delta frequency ISI peak (Fig. 1i, j). We found that 81% of ChIs exhibited delta-rhythmic behavior, compared to only 36% of SYNs (Fig. 1k, l). While the overall firing rate of ChI and SYN populations were similar (Fig. 1m), delta-rhythmic neurons as a population had a significantly lower firing rate than non-delta neurons (Fig. 1n). While this effect was less pronounced in ChIs (Fig. 1o), delta-rhythmic SYNs, in particular, had significantly lower firing rates than non-delta SYNs (Fig. 1p). For both ChIs and SYNs, delta-rhythmic neurons had inter-burst firing rates at delta frequency as expected (ChIs: 2.28 ± 0.034 Hz, SYNs: 2.27 ± 0.039 Hz).

### Delta rhythmic striatal neurons exhibit prominent subthreshold membrane voltage (Vm) delta oscillations that organize spike timing

**Fig. 2:**
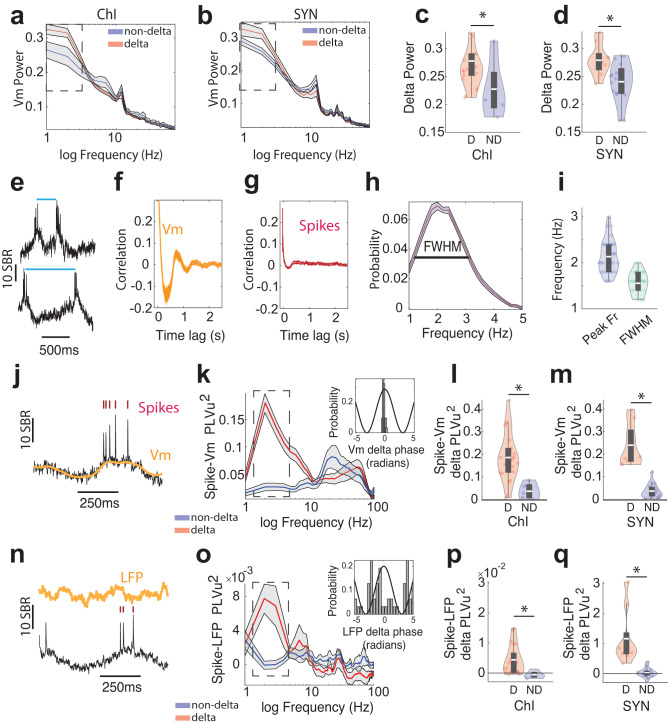
Subthreshold membrane voltage (Vm) delta rhythm structures spike timing and Vm. **a**, **b** Population Vm spectral power of (**a**) delta-rhythmic ChIs (red, *n* = 22) and non-delta ChIs (blue, *n* = 5) and of (**b**) delta-rhythmic SYNs (red, *n* = 9) and non-delta SYNs (blue, *n* = 16). Boxes highlight delta frequencies. **c** Delta power (1–4 Hz) for delta-rhythmic (D) and non-delta (ND) ChIs (independent *t*-test, *p* = 0.047, *n* = 27, df = 25). **d** Same as **c**, but for SYNs (independent *t*-test, *p* = 0.006, *n* = 25, df = 23). **e** Illustration of delta-cycle length variability in an example ChI. **f**, **g** The population (**f**) Vm and (**g**) spike autocorrelogram for delta-rhythmic ChIs (*n* = 22). **h** Population-averaged instantaneous Vm delta-frequency distribution (1–6 Hz was used to better capture the variations in instantaneous delta-frequencies). FWHM, full-width-at-half-maximum. **i** Quantification of peak delta frequency and FWHM of delta-frequency distribution (*n* = 31). **j** An example striatal neuron’s Vm filtered at 1–4 Hz (yellow) and spikes (red ticks). **k** The squared phase-locking value (PLVu^2^) of spikes to Vm across frequencies for delta-rhythmic (red) and non-delta neurons (blue). Inset: the polar histogram of an example delta-rhythmic neuron’s preferred spike phase to Vm delta. **l** Spike-Vm PLVu^2^ around peak delta frequency (2–3 Hz) in delta-rhythmic (D, red) versus non-delta ChIs (ND, blue) (independent *t*-test, *p* = 0.005, *n* = 27, df = 25). **m** Same as **l**, but for SYNs (independent *t*-test, *p* = 3.08 × 10^−8^, *n* = 25, df = 23). **n** Example voltage trace of a striatal neuron with spikes (red ticks) and corresponding LFP (yellow). **o** PLVu^2^ of spikes to LFP across frequencies for delta-rhythmic (red) and non-delta neurons (blue). Inset: the polar histogram of delta-rhythmic neurons’ preferred spike phase (mean angle) to LFP delta oscillations. **p** Spike-LFP PLVu^2^ in the delta frequency range between delta-rhythmic ChIs and non-delta ChIs (independent *t*-test, *p* = 0.032, *n* = 27, df = 25). **q** Same as **p**, but for SYNs (independent *t*-test, *p* = 4.84 × 10^−5^, *n* = 25, df = 23). All shaded regions around line plots represent the standard error of mean. Quantifications are visualized as violin plots with the outer shape representing the data kernel density and a box-and-whisker plot (box: interquartile range, whiskers: 1.5x interquartile range, white line: mean). All statistical tests are two-sided. Source data are provided as a Source Data file.

Spike timing critically depends on membrane voltage dynamics, and we noticed spike bursting in delta-rhythmic neurons was often associated with prominent somatic Vm depolarizations at delta frequencies (Fig. 1c). Indeed, the time-averaged Vm wavelet power of delta-rhythmic neurons had a significantly higher delta power than non-delta neurons (Fig. 2a, b), for both ChI and SYN populations (Fig. 2c, d). Further, we found substantial variability of the peak delta frequency over time (Fig. 2e) resulting in weak delta time-scale autocorrelograms (Fig. 2f, g). Since delta frequencies are slow, slight variation within the delta frequency range could result in large differences in cycle length ranging from around 0.25 to 1 s. We thus further examined the distribution of instantaneous delta frequency across delta cycles (peak frequency at 2.13 ± 0.36 Hz) and quantified the delta frequency variability using the full-width-at-half-maximum of the frequency distribution (FWHM, 1.55  ± 0.24 Hz, Fig. 2h, i). The large variation in instantaneous delta frequencies could explain the lack of obvious spike rhythmicity of ChIs in previous extracellular recording studies that characterized putative ChIs as tonically active neurons (TANs) with wide extracellular spike waveforms^34,43,44^.

To further investigate how the delta oscillations we observed relate to spike timing and higher frequency oscillations, we calculated the squared phase locking value (PLVu^2^; unbiased by the number of spikes, see Methods) of spikes relative to Vm (Fig. 2j, k) and LFP oscillations (Fig. 2n, o). For both ChIs and SYNs, spike-Vm PLVu^2^ at delta frequency peaks (2–3 Hz) was significantly higher for delta-rhythmic neurons than non-delta neurons (Fig. 2l, m). Further, while LFP oscillations in the striatum are weak due to the lack of generally organized dendritic configuration among striatal neurons, we found spike-LFP PLVu^2^ (Fig. 2n, o) to be the strongest in the same 2–3 Hz delta frequency range as for Vm for both ChIs and SYNs. As with Vm, spikes in delta neurons were significantly more phase-locked (higher PLVu^2^) than non-delta neurons to these LFP delta oscillations (Fig. 2p, q). As breathing and other biological motion may also occur around delta frequencies, we examined whether the observed spike phase locking to Vm and LFP delta may be related to image motion that is inherent in fluorescence imaging studies. We computed PLVu^2^ of spikes to image motion, calculated as the imaging frame shift during motion correction (details in Methods). We found that spike-image motion PLVu^2^ was generally weak, with no difference between delta-neurons and non-delta neurons, and no noticeable peaks around the delta frequencies (Supplementary Fig. 4). Thus, spiking coordination with Vm or LFP delta rhythms cannot be explained by potential image motion. Taken together, these results demonstrate that many striatal neurons, in particular ChIs, exhibit prominent Vm delta oscillations, which organize spike timing that is also coordinated with network-level LFP delta oscillations.

### The phase of Vm delta oscillations is coupled to beta power modulation in both Vm and LFP

**Fig. 3:**
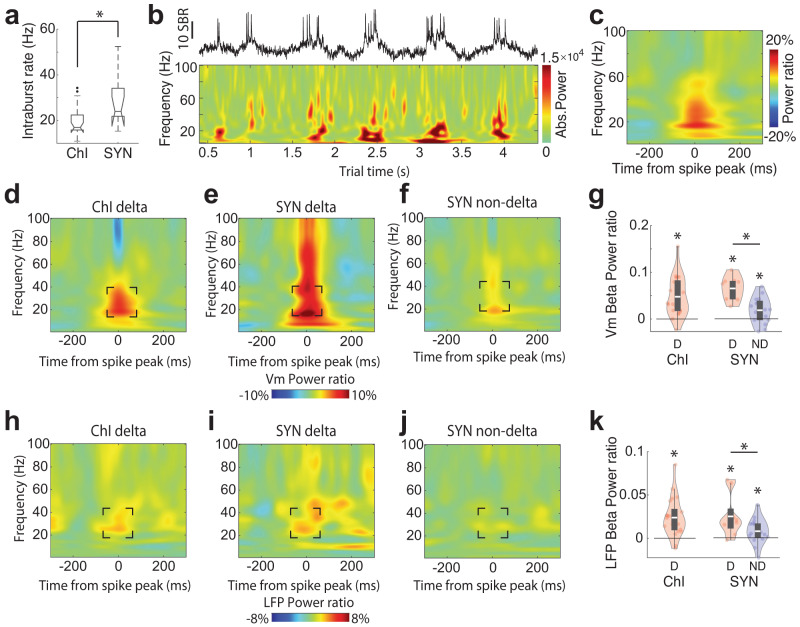
Subthreshold Vm delta rhythm structures Vm and LFP beta power. **a** Mean intraburst firing frequency for delta-rhythmic ChIs and SYNs (independent *t*-test, *p* = 0.007, *n* = 31, df = 29). **b** Example voltage trace of a delta-rhythmic striatal neuron with corresponding Vm wavelet power spectrum shown below. **c** Mean normalized Vm spectral power aligned to the peak of all spikes in the example neuron shown in **b**. **d**–**f** Vm power around spike peak relative to pre-spike period (−200 to −100 ms) for (**d**) delta-rhythmic ChIs (*n* = 21), (**e**) delta-rhythmic SYNs (*n* = 9), and (**f**) non-delta rhythmic SYNs (*n* = 16). **g** Quantification of Vm power around spike peak relative to pre-spike period at beta-frequencies (20–40 Hz) for delta-rhythmic (D, red) and non-delta (ND, blue) ChIs (left), and SYNs (right). Vm beta power at spike peak was significantly higher than pre-spike period in all groups (all one-sample *t*-test, ChI D: *p* = 1.7 × 10^−5^, df = 21; SYN D: *p* = 5.3 × 10^−5^, df = 8; SYN ND: *p* = 0.015, df = 15). Delta-rhythmic SYNs showed greater modulations than non-delta SYNs (independent *t*-test SYN D vs SYN ND: *p* = 3.64 × 10^−4^, *n* = 25, df = 23). **h**–**j** Spike-aligned LFP power (relative to pre-spike period) for (**h**) delta-rhythmic ChIs (*n* = 21), (**i**) delta-rhythmic SYNs (*n* = 9), and (**j**) non-delta SYNs (*n* = 16). **k** Quantification of LFP power around spike peak relative to pre-spike period at beta frequencies for delta-rhythmic (red) and non-delta (blue) ChIs (left), and SYNs (right). LFP beta power at spike peak was significantly higher than pre-spike period in all groups (all one-sample *t*-test, ChI D: *p* = 8.9 × 10^−5^, *n* = 22, df = 21; SYN D: *p* = 0.0147, *n* = 9, df = 8; SYN ND: *p* = 0.04, *n* = 16, df = 15), and delta-rhythmic SYNs showed greater modulations than non-delta SYNs (independent *t*-test SYN D vs SYN ND: *p* = 0.037, *n* = 25, df = 23). Quantifications are visualized as violin plots with the outer shape representing the data kernel density and a box-and-whisker plot (box: interquartile range, whiskers: 1.5x interquartile range, white line: mean). All statistical tests are two-sided. Source data are provided as a Source Data file.

In delta rhythmic ChIs and SYN neurons, we found that the intraburst firing rate of delta-rhythmic neurons was centered around the beta frequency range (ChIs: 21 ± 1.5 Hz, SYNs: 27.3 ± 3.57 Hz; Fig. 3a), suggesting that delta-rhythmic firing patterns might be associated with beta rhythms. When we aligned Vm wavelet spectrum power to individual SomArchon voltage traces, we noticed that spikes were indeed accompanied by an increase in Vm beta-band (20–40 Hz) power (Fig. 3b, c). Vm beta-band power was significantly higher around spikes in delta-rhythmic neurons for both ChI and SYN populations (Fig. 3d, e, g), though the increase is much less pronounced in non-delta SYNs (Fig. 3f, g). Non-delta ChIs were not examined due to low neuron numbers. To understand whether the increase in Vm beta power was related to spikes occurring at beta-frequencies within a burst, we separated the Vm delta cycles into periods that included spikes versus those without, and found that Vm beta power remained elevated at the peaks of delta oscillations even in the absence of spiking (Supplementary Fig. 5). Thus, Vm delta-beta cross-frequency coupling is an intrinsic feature of Vm dynamics and is not due to delta-rhythmic spike bursting.

A similar analysis using LFP spectrum power showed that spikes in delta-rhythmic neurons were also coupled to an increase in LFP beta power for both ChI and SYN populations (Fig. 3h, i, k), though less prominent in non-delta rhythmic neurons (Fig. 3j, k). Together, these results demonstrate that cellular Vm delta-beta cross-frequency oscillations organize beta frequency synchronization at the single neuron membrane voltage level. Further, spiking in striatal neurons that exhibit delta-rhythmicity are selectively coupled to prominent LFP delta and beta oscillations, suggesting that synchronization among delta-rhythmic neurons contributes to fluctuations in striatal LFP delta and beta power.

### Delta-rhythmic neurons are uniquely activated during high-speed movement and at movement transitions

**Fig. 4:**
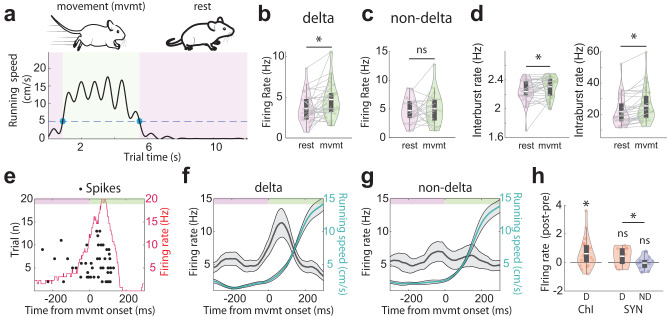
Delta-rhythmic neurons, but not non-delta neurons, exhibit locomotion-dependent spiking. **a** An example locomotion speed recording (black line) while an animal was voluntarily running on the treadmill. The dashed blue line indicates the threshold (5 cm/s) used to classify periods of rest (pink) and movement (green). Blue dots mark the onset and offset of movement periods. The schematic resting mouse was adapted from 10.5281/zenodo.3925949 and running mouse from 10.5281/zenodo.3925901. **b** Firing rates in delta-rhythmic neurons during rest versus movement (paired *t*-test, *p* = 0.0026, *n* = 31, df = 30). **c** Same as **b**, but for non-delta neurons (paired *t*-test, *p* = 0.556, *n* = 21, df = 20). **d** Left: Interburst firing rates of delta-rhythmic neurons during rest versus movement (paired *t*-test, *p* = 0.005, *n* = 31, df = 30). Right: Intraburst firing rates of delta-rhythmic neurons during rest versus movement (paired *t*-test, *p* = 1.7 × 10^–5^, *n* = 31, df = 30). **e** Spike raster plot aligned to movement onset for an example ChI. Black points are spike times, and the red line indicates the average firing rate across all movement onsets. **f**, **g** Population firing rate aligned to movement onset for (**f)** delta-rhythmic neurons (*n* = 31) and (**g**) non-delta neurons (*n* = 21). Turquoise lines indicate running speed. **h** Quantification of movement onset-related firing rate changes calculated as post-onset (0 to 120 ms) minus pre-onset (−120 to 0 ms). Only delta-rhythmic (D) ChIs increased their firing rates at onset (all one-sample *t*-test, ChI, D: *p* = 0.019, *n* = 21, df = 20, one neuron excluded due to lack of movement data; SYN, D: *p* = 0.099, *n* = 9, df = 8; SYN, non-delta (ND): *p* = 0.53, *n* = 16, df = 15). Delta-rhythmic SYNs had significantly stronger firing enhancement than non-delta SYNs (independent *t*-test SYN D vs SYN ND: *p* = 0.041, *n* = 25, df = 23). The shaded regions around line plots represent the standard error of the mean. Quantifications are visualized as violin plots with the outer shape representing the data kernel density and a box-and-whisker plot (box: interquartile range, whiskers: 1.5x interquartile range, white line: mean). All statistical tests are two-sided. Source data are provided as a Source Data file.

As striatal neurons and striatal LFP are modulated by various aspects of locomotion^35,45–47^, we examined whether delta rhythmic vs. non-delta rhythmic neurons exhibited different locomotion-related responses. To assess locomotion-related responses, we classified stages of motor output into movement bouts (defined as treadmill speed ≥5 cm/s), resting bouts (treadmill speed <5 cm/s), and movement onset and offset transitions between these movement bouts (Fig. 4a). We found that delta-rhythmic neurons significantly increased their firing rates as a population during movement compared to during rest, while non-delta neurons did not change their firing rates (Fig. 4b, c). Interestingly, delta-rhythmic neurons’ interburst and intraburst spike rates both increased during movement, as compared to resting periods (Fig. 4d). When we categorized these neurons based on cell type, we found that SYNs exhibited more heterogeneity in their movement responses than ChIs, and thus SYNs as a population were insensitive to movement speed while ChIs as a population increased their firing rate during movement (Supplementary Fig. 6).

Transitions in movement speed have been shown to critically depend on the striatum and are sensitive to striatal dopamine changes^35,36,46^. We thus examined the responses of delta-rhythmic and non-delta rhythmic neurons around movement transitions. An example of a delta-rhythmic ChI responsive to movement onset is shown in Fig. 4e. We found that delta-rhythmic neurons as a population significantly increased their firing rates around movement onset (Fig. 4f). Non-delta neurons, in contrast, showed no change in firing rate around movement onset (Fig. 4g). By further examining ChI and SYN subgroups within the delta and non-delta populations, we found both delta-rhythmic ChIs and SYNs significantly increased their firing rates around movement onset, while non-delta-rhythmic SYNs did not show movement onset-related firing rate modulations (Fig. 4h and Supplementary Fig. 7a–c), similar to their differential responses during sustained movement periods (Fig. 4b, c). Due to the low number of non-delta ChIs and the limited number of movement transitions, we did not perform this analysis on the non-delta ChI group. Finally, none of the three groups’ firing rates changed significantly around movement offset (Supplementary Fig. 7d–f). Together, these results provide direct experimental evidence that delta-rhythmic neurons, both ChIs and SYNs, but not non-delta rhythmic neurons, selectively encode movement onset transitions and sustained movement.

### Delta-rhythmic ChI and SYN spiking is differentially associated with LFP delta, beta, and gamma rhythms during movement

**Fig. 5:**
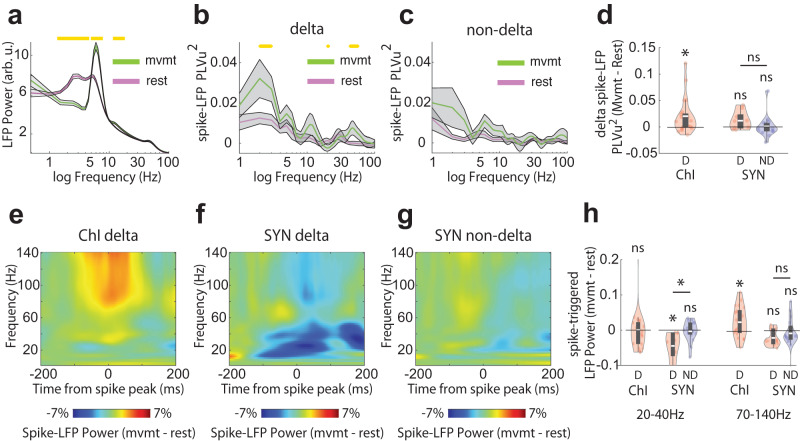
Delta-rhythmic ChIs and SYNs exhibit distinct, locomotion-dependent temporal relationships to LFP delta, beta, and gamma oscillations. **a** LFP power during rest (pink) and movement (green). **b** Spike-LFP PLVu^2^ during rest (pink) and movement (green) for delta-rhythmic neurons. Yellow dots mark a significant difference between rest and movement periods (*p* < 0.05). **c** Same as **b**, but for non-delta neurons. **d** Quantification of spike-LFP PLVu^2^ difference at delta frequencies (1–4 Hz) between rest and movement. Only delta-rhythmic (D) ChIs (red) exhibit enhanced spike-LFP PLVu^2^ during movement (all one-sample *t*-test, ChI D: *p* = 0.008, *n* = 21, df = 20; one neuron was excluded due to the lack of movement data; SYN D: *p* = 0.068, *n* = 9, df = 8; SYN ND: *p* = 0.91, *n* = 16, df = 15), while delta-rhythmic SYNs have similar firing rate modulation as non-delta SYNs (independent *t*-test SYN D vs SYN ND: *p* = 0.09, *n* = 25, df = 23). **e**–**g** Population spike-aligned LFP spectral power difference between rest and movement for (**e**) delta-rhythmic ChIs, (**f**) delta-rhythmic SYNs, and (**g**) non-delta SYNs. **h** Quantification of normalized LFP power difference between rest and movement for delta-rhythmic ChIs and SYNs (red), and non-delta SYNs (blue) in the beta range (20–40 Hz; One ChI was excluded due to the lack of movement data. Within-group comparison, all one-sample *t*-test, ChI D: *p* = 0.97, *n* = 21, df = 20; SYN D: *p* = 0.004, *n* = 9, df = 8; SYN ND: *p* = 0.4, *n* = 16, df = 15; Between-group comparison, independent *t*-test, SYN D vs SYN ND: *p* = 0.005, *n* = 25, df = 23) and high-gamma range (70–100 Hz, Within-group comparison, all one-sample *t*-test, ChI D: *p* = 0.019, *n* = 21, df = 20, SYN D: *p* = 0.054, *n* = 9, df = 8; SYN ND: *p* = 0.51, *n* = 16, df = 15; Between-group comparison, independent *t*-test, SYN D vs SYN ND: *p* = 0.45, *n* = 25, df = 23).All shaded regions around line plots represent the standard error of the mean. Quantifications are visualized as violin plots with the outer shape representing the data kernel density and a box-and-whisker plot (box: interquartile range, whiskers: 1.5x interquartile range, white line: mean). All statistical tests are two-sided. Source data are provided as a Source Data file.

Given that delta-rhythmic neurons exhibited stronger spike phase locking to Vm and LFP delta oscillations (Fig. 2k, o), we next examined whether locomotion-related spiking was linked to network delta rhythmicity measured as LFP oscillations. We found that striatal LFP power at delta frequency (2–4 Hz) was elevated during rest, while narrow-band theta (6–8 Hz) and alpha (10–11 Hz) oscillations were elevated during movement (Fig. 5a). Despite the dominant peak in the theta range of LFP power during movement, the spike-LFP PLVu^2^ at delta frequency, but not theta frequency, was significantly increased during locomotion for delta-rhythmic neurons (Fig. 5b), driven specifically by increased PLVu^2^ of delta-rhythmic ChIs (Fig. 5d). This suggests that LFP delta power, while not as striking as LFP theta power, nevertheless showed a movement-specific association to striatal ChIs spiking in the delta frequency range. Neither delta-rhythmic nor non-delta rhythmic SYNs exhibited significant changes in spike-LFP delta PLVu^2^ during movement (Fig. 5c, d). The unique temporal relationship of ChI spiking with LFP delta oscillations highlights an important role of ChIs in influencing striatal network LFP delta rhythmicity.

We next analyzed whether spike-LFP relationships were differentially modulated by movement conditions. We calculated the difference of LFP spectrum power aligned to spikes that occurred during movement versus rest (Fig. 5e–g). Delta-rhythmic ChI spiking was associated with significantly increased LFP high-gamma (70–140 Hz), but not beta (20–40 Hz) power, during movement compared to rest (Fig. 5e, h and Supplementary Fig. 8a, d). Thus, even though Vm and LFP beta power increased around spikes (Fig. 3d, h, g, k), LFP beta oscillations accompanying ChI spiking were not specific to movement. In contrast, delta-rhythmic SYN spiking was associated with a significant decrease in beta power during movement, which started ~100 ms before the spike and lasted up to ~200 ms after (Fig. 5f, h and Supplementary Fig. 8b, e). The reduction in LFP beta power around SYN spiking is consistent with previous studies reporting overall reductions in LFP beta power during movement^46,48^. No significant movement-dependent differences in LFP oscillations were detected around spikes of non-delta SYNs (Fig. 5g, h and Supplementary Fig. 8c, f).

Finally, we found a selective association of LFP high-gamma power with delta-rhythmic ChI spiking, but not SYN spiking, during movement (Fig. 5e, h) highlighting a role of ChI spiking in synchronized striatal neuron activation, which is supported by the unique anatomical features of ChIs that form extensive synaptic connections with striatal neurons.

### Delta-rhythmic neurons spike in coordination with movement stepping cycles

**Fig. 6:**
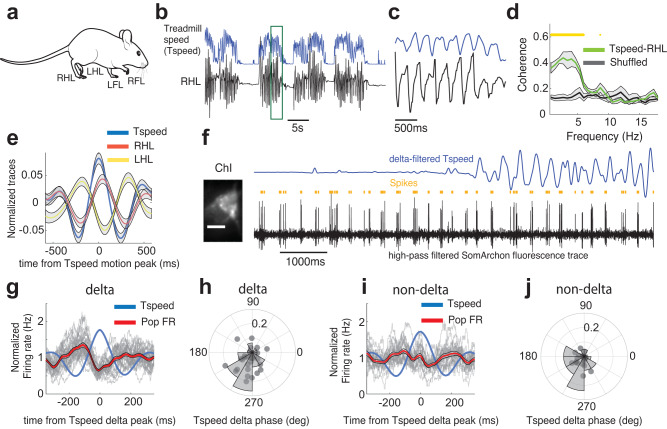
Delta components of locomotion speed are linked to limb stepping cycles and delta-rhythmic neuron spiking. **a** Illustration of mouse locomotion with the four limbs labeled. The schematic mouse is adapted from 10.5281/zenodo.3925915. **b** A single trial example of treadmill speed (Tspeed, blue) and simultaneously measured right hindlimb (RHL) position (black). **c** Zoom-in of the time window depicted by the green box in **b**. **d** Population coherence between treadmill speed and right hindlimb (RHL) position. Yellow dots mark a significant difference between rest and movement (bootstrapping, *p* < 0.05, *n* = 4 mice). **e** RHL and LHL positions aligned to the peak of the delta-frequency component of treadmill speed. **f** A single trial example of a delta-rhythmic ChI’s membrane voltage (high-pass filtered, black) with spike times (yellow ticks) and accompanying delta-frequency (1–6 Hz) component of treadmill speed (blue). Left, averaged SomArchon fluorescence image of the example neuron. Scale bar, 15 µm. **g** Normalized firing rate of delta-rhythmic neurons aligned to the peak of the delta-frequency component of treadmill speed. The firing rate is normalized by the mean firing rate of a given neuron (*n* = 30, only neurons with a minimum of 10 delta-peak triggered windows were included). Gray lines represent individual neurons, the red line is the population average. **h** Polar histogram and scatter plot of preferred spiking phase of delta-rhythmic neurons (*n* = 30, neurons with a minimum of 10 delta-peak triggered windows were included) relative to the delta-frequency component of treadmill speed. **i**, **j** Same as **g**, **h**, but for non-delta neurons (*n* = 21). All shaded regions around line plots represent the standard error of the mean. Quantifications are visualized as violin plots with the outer shape representing the data kernel density and a box-and-whisker plot (box: interquartile range, whiskers: 1.5x interquartile range, white line: mean). All statistical tests are two-sided. Source data are provided as a Source Data file.

Stepping movement in humans and mice occurs in the delta frequency range^1–4^, and temporally precise neuronal spiking at delta frequencies in the motor cortex, cerebellum, and thalamus has been linked to rhythmic stepping movement^4,10–12^. Intrigued by the frequency similarity between striatal neurons’ delta rhythmicity and mouse stepping cycles, we further analyzed mouse stepping during movement. We found that during locomotion periods, movement speed recorded by our treadmill contained delta-rhythmic fluctuations similar to the delta frequency range observed in Vm. To evaluate whether delta-frequency movement-speed fluctuations detected by our treadmill corresponded to stepping cycles, we performed simultaneous video tracking of limb movements and treadmill speed recordings (Fig. 6a–c). We found that limb movement was highly coherent with the fluctuations in movement speed at delta-frequencies captured by our treadmill recordings (Fig. 6c, d). Specifically, the movement of the right and left hind limbs (RHL and LHL, respectively) exhibited strong phase locking to the delta-frequency component of the treadmill speed, with a time lag between the two hind limbs, reflective of the alternating limb movement patterns within each stepping cycle (Fig. 6d, e). Thus, the delta-frequency treadmill speed fluctuations we observed captured the animal’s delta-rhythmic stepping cycles.

**Fig. 7:**
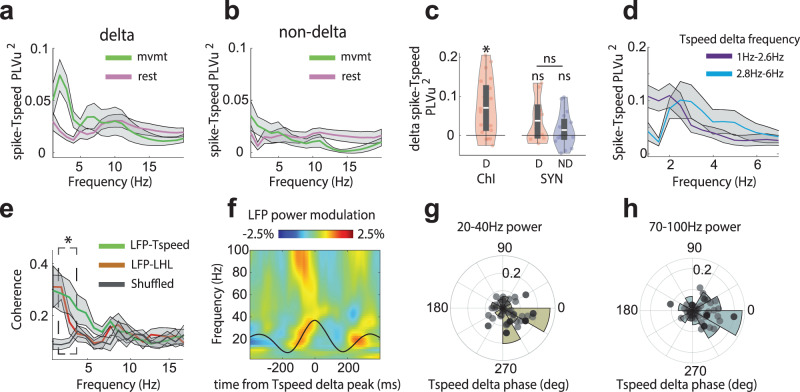
Delta-patterned locomotion orchestrates delta-rhythmic striatal neuron spiking and high-frequency rhythms. **a** Spike phase-locking value (PLVu^2^) relative to treadmill speed (Tspeed) during rest and movement for delta-rhythmic neurons (*n* = 30, neurons with a minimum of 10 spikes during rest and movement respectively). **b** Same as in **a**, but for non-delta neurons (*n* = 21). **c** Quantification of spike-Tspeed PLVu^2^ relative to delta-filtered Tspeed (2–3 Hz) during rest vs movement for delta-rhythmic ChIs, delta-rhythmic SYNs, and non-delta SYNs. Only delta-rhythmic ChIs exhibited significantly enhanced spike-Tspeed PLVu^2^ during movement (all one-sample *t*-test, ChI D: *p* = 1.85 × 10^−4^, *n* = 21, df = 20; SYN D: *p* = 0.08, *n* = 9, df = 8; SYN ND: *p* = 0.18, *n* = 16, df = 15). Delta-rhythmic SYNs and non-delta SYNs showed no differences (independent *t*-test SYN D vs SYN ND: *p* = 0.26, *n* = 25, df = 23). **d** Spike-Tspeed PLVu^2^ for delta-rhythmic neurons (*n* = 21, only neurons with >10 spikes during movement period were included) for treadmill speed at 1–2.6 Hz (purple) or 2.8–6 Hz (blue). **e** PLVu^2^ for LFP-Tspeed and LFP-left hindlimb (LHL) during movement, with shuffled LFP-Tspeed as reference (gray) (paired *t*-test at 2–3 Hz delta-frequency peak, *n* = 5, LFP-Tspeed, *p* = 3.3 × 10^−4^, LFP-LHL, *p* = 0.074). **f** LFP power modulation aligned to the peak of the delta-frequency component of treadmill speed and normalized by its mean power over the time window. **g** Polar histogram and scatter plot depicting the preferred phase of beta power (20–40 Hz) relative to the delta-frequency component of treadmill speed. Each dot corresponds to a recording session (Omnibus test for non-uniformity, *p* = 0.012, *n* = 59). **h** Same as **g**, but for high-gamma power (70–100 Hz, Omnibus test for non-uniformity, *p* = 0.005, *n* = 59). All shaded regions around line plots represent the standard error of the mean. Quantifications are visualized as violin plots with the outer shape representing the data kernel density and a box-and-whisker plot (box: interquartile range, whiskers: 1.5x interquartile range, white line: mean). All statistical tests are two-sided. Source data are provided as a Source Data file.

Since spike times were strongly modulated by Vm delta oscillations, we next examined whether delta-rhythmic neurons’ spiking activity was coupled to stepping cycles, measured as the delta-frequency component of the treadmill speed (Fig. 6f). As a population, delta neurons significantly increased their firing rates prior to the peak of treadmill speed delta, as opposed to non-delta neurons (Fig. 6g, i). Delta-rhythmic neurons generally spiked in the rising phase (−1.93 ± 0.97 radians, mean ± SD) of the delta-frequency component of treadmill speed (Fig. 6h). Non-delta rhythmic neurons showed a similar phase preference (−2.2 ± 0.98 radians, mean ± SD), despite having weak spike phase locking (Fig. 6j). Indeed, we found that spikes in delta-rhythmic neurons, but not non-delta neurons, exhibited significant phase locking (PLVu^2^) to the delta-frequency component (2–3 Hz) of mouse locomotion speed during movement (Fig. 7a–c). Separating delta-rhythmic ChI and SYN populations revealed that this effect was driven mainly by delta-rhythmic ChIs, but not delta-rhythmic SYNs (Fig. 7c). Finally, as the instantaneous delta frequencies during stepping varied (mean ± SD, 2.71 ± 1.23), we examined the delta frequencies below versus above the mean. Most remarkably, delta-rhythmic neurons remained phase locked across different ranges of stepping frequencies (Fig. 7d).

To further understand the temporal relationship between delta-rhythmic neuron spiking and stepping cycles, we aligned the firing rate to the peak of the first delta cycle within a movement bout. We found that delta-rhythmic neurons significantly increased their firing rate before the first delta peak around movement onset, with the firing rate preceding stepping movement by about 200 ms (Supplementary Fig. 9a). Similar firing rate changes proceeded the last delta peak around movement offset (Supplementary Fig. 9b). Thus, stepping-patterned delta-rhythmic neurons’ spiking persists throughout the locomotion bouts.

Finally, we found that striatal LFPs were highly coherent to both treadmill speed and left hindlimb movement in the stepping-related delta frequency range (Fig. 7e). Further, LFP beta (20–40 Hz) and high-gamma (70–100 Hz) power were modulated by the stepping cycle (Supplementary Fig. 10) and the delta-frequency component of the treadmill speed, peaking at a similar phase as the spikes in delta-rhythmic neurons (Fig. 7f–h). Thus, striatal LFP beta and high-gamma power are nested within the delta-rhythmic stepping cycle. Stepping-modulated LFP high-gamma power is most likely due to increased neural activities, as high-gamma oscillations have been broadly associated with elevated spike rates. Since spiking and Vm delta rhythmicity in delta-rhythmic neurons are coupled to Vm and LFP beta oscillations regardless of movement conditions, the observed stepping-modulated LFP beta oscillations reflect the synchronization of delta-rhythmic neurons’ Vm with stepping cycles. Together, our results demonstrate a prominent role of delta-rhythmic striatal neuron spiking and striatal LFPs to influence mouse stepping cycles and organize higher frequency beta and gamma oscillations, providing evidence for a functional role of basal ganglia delta-rhythmic dynamics in movement patterning.

## Discussion

Synchronized neural activity at delta frequencies is broadly observed in cortical, subcortical, and spinal motor circuits during delta-rhythmic voluntary movement^1–6^. Network delta rhythms have been proposed to serve as a temporal coordination mechanism to organize higher frequency rhythms and spiking^5,6,49^. To understand how cellular dynamics of individual neurons support network delta rhythmicity during locomotion, we performed membrane voltage imaging from genetically-defined striatal cholinergic interneurons (ChIs) and non-specific striatal cells expressing the generic neuronal marker synapsin (SYNs) using the genetically encoded voltage indicator SomArchon^41^. We extracted voltage-dependent SomArchon fluorescence and analyzed spiking and subthreshold membrane voltage (Vm) at the soma of individual ChIs and SYNs, while mice voluntarily locomoted on a treadmill. We found that many striatal neurons, particularly ChIs, have prominent cellular Vm oscillations and spiking at delta frequencies during both rest and movement. Not only did Vm delta oscillations pace spiking output at beta-frequencies, but they were also coupled to LFP delta and beta oscillations, and tightly phase locked to animals’ delta-rhythmic stepping cycles. Thus, our study provides direct experimental evidence that delta rhythmicity within single striatal neuron membrane voltage plays an important role in the patterning of movement.

ChIs, though representing only 1–2% of the striatal cell population, can powerfully modulate striatal networks via their extensive synaptic arborizations that connect broadly throughout the striatal network^30–32,43^. A remarkable feature of the ChIs we observed was the sustained Vm oscillations and spike inter-bursting intervals in the delta range (1–4 Hz), during both movement and resting conditions. Given ChIs exhibit similar intrinsic delta-rhythmic Vm depolarizations and spike bursting in vitro without any synaptic inputs^33,34,44^, it is likely that intrinsic biophysical mechanisms are critical for shaping the ChI Vm delta rhythms observed here in awake animals, regardless of locomotor conditions. However, ChIs also receive broad cortical and thalamic inputs^50^ and many of these inputs exhibit delta-frequency rhythmicity that could contribute to the observed ChI Vm delta oscillations^4^. Furthermore, we also noted the presence of Vm delta oscillations in a subset of non-specific striatal neurons (SYNs), estimated to be primarily SPNs. Since SPNs do not demonstrate an intrinsic resonance frequency preference^33^, the observed cellular delta rhythmicity in SYNs is likely due to synaptic inputs rather than intrinsic biophysical mechanisms.

We also found that ChI spiking is time-locked to movement onset, and tracks the animal’s stepping cycle in the 1–4 Hz delta frequency range. Studies have reported that certain neurons in the motor cortex, thalamus, and cerebellum have spikes phase locked to LFP delta oscillations^10–14,51^, and projections from these areas to the striatum exhibit stepping cycle-dependent activity^10–14^. Thus, the movement-dependent entrainment of cellular delta rhythms observed in ChIs likely originates from movement-related synaptic inputs.

Not only do ChIs receive extensive dopaminergic inputs from the substantia nigra pars compacta (SNc) and glutamatergic inputs from broad cortical and thalamic regions, but ChIs can directly modulate these inputs via nicotinic acetylcholine receptors (nAchRs) expressed on these input axon terminals^30–32^. Indeed, fast dopaminergic signaling at SNc axon terminals in the striatum exhibit delta frequency modulation^52^. Since ChI activation can powerfully excite dopaminergic and glutamatergic terminals via nAchRs, it is possible that delta-rhythmic spiking of ChIs paces striatal output through indirect action on these input axonal terminals, in addition to SPNs. SPNs additionally express metabotropic acetylcholine receptors (mAChRs), and activation of ChIs could increase SPN activity, particularly in D2-SPNs that express mainly excitatory mAChR1, which could promote striatal LFP beta oscillations^40^. ChIs have been shown to promote coordination between SPNs, and the elevation of striatal cholinergic tone increases LFP beta and gamma oscillations leading to movement inhibition^35,39,40,46^. We detected an increase in LFP beta power associated with spiking not only in delta-rhythmic ChIs, but also in delta-rhythmic SYNs, dominated by SPNs, in the absence of movement. It is likely that the LFP beta power around delta-rhythmic SYN spiking is due to direct pacing by ChIs whose spikes are associated with both Vm and LFP beta rhythms.

In addition to rhythmic stepping, other potential rhythmic movements include respiration, sniffing, and whisking^53–55^ which could produce widespread effects on subcortical and cortical motor circuits. The reported respiration frequency in mice is commonly in the theta range (3–10 Hz), particularly during locomotion. Recent studies found no temporal relationship between respiration and stepping cycle^56^, suggesting that the Vm delta rhythmicity observed here is unlikely to be linked to the respiration cycle. Whisking, which often occurs at a higher speed of 10 Hz or more, has been shown to be phase coupled to strides during certain behavioral conditions^55^. It is thus possible that some of the Vm delta rhythmicity we observed in striatal neurons is related to other rhythmic movement that is coupled to stepping.

The central pattern generator circuits in the spinal cord are critical for producing autonomous rhythmic motor patterns that usually occur in the delta frequency range^8,9^. However, the spinal central pattern generator receives substantial supra-spinal control from diverse brainstem circuits, the cerebellum, thalamus, motor cortex, and basal ganglia^7–9^. While stepping cycle modulated activity has been well documented in the cerebellum and motor cortex^10–14^, the basal ganglia has been traditionally associated with action selection, motor learning, and various locomotion features, rather than motor patterning^35,46,57,58^. Recently, it has been shown that the subthalamic nucleus (STN) of the basal ganglia exhibits stepping cycle-dependent activity^59,60^. Further, striatal neurons code for movement vigor^61^ and Parkinsonian patients tend to have gait disturbances including changes in their stepping frequency^3,15–18^, suggesting that the basal ganglia may also influence stepping dynamics. In fact, we detected prominent cellular membrane voltage delta rhythmicity in ChIs regardless of movement conditions, which along with ChI’s powerful influence on striatal circuitry, suggests that ChIs may not only modulate or pace movement patterns, but provide temporal patterning for a wide variety of sensorimotor and cognitive functions.

Delta-rhythmic population activity in the motor cortex and thalamus has been linked to population dynamics during rhythmic and non-rhythmic motor behaviors^4,5,14^. The sustained delta-rhythmic patterning of striatal activity might play a key role in supporting the coordination of the cortico-basal ganglia-thalamic circuits during movement, which can interact with spinal circuits via frequency-dependent circuit coupling. Finally, striatal neurons are sensitive to various aspects of movement, including movement transitions, speed, direction, and complex moment-to-moment behavioral kinematics^35,46,57,58^. It would be informative to examine how individual striatal neuronal membrane voltage dynamics relate to various movement aspects in addition to movement patterning in future studies.

The functional properties of striatal tonically active neurons (TAN), assumed to largely correspond to cholinergic interneurons, have been extensively examined using extracellular electrophysiology in rodents and primates^43^. However, strong spike delta rhythmicity, as observed here, has not been reported. While we found clear subthreshold delta rhythms in the membrane potential that predicted spike timing, we found no delta rhythmicity in the spike autocorrelogram and only weak delta rhythmicity in the Vm autocorrelogram due to the large cycle or frequency variability within the delta range. This quasi-periodic property is in line with delta-rhythmic pattern generation dynamics reported in the motor cortex^5^.

We found that beta rhythms in both Vm and LFP signals were nested within the delta-rhythm, with a preferred phase similar to the spiking preference of ChIs and SYNs. Stepping cycle nested LFP beta and gamma power has also been observed in the human STN^59,60^, suggesting that during locomotion, delta rhythms in the cortico-basal ganglia circuit are coordinated with patterned stepping movement, and beta and gamma oscillations across structures are nested within delta cycles. Further, we observed that neurons with Vm delta oscillations exhibited stronger spike-associated beta power than non-delta neurons, highlighting that striatal delta and beta rhythms are closely linked at the cellular level, which is consistent with reported delta-beta cross-frequency coupling in the motor thalamus^48^ and more generally, across the cortico-basal-ganglia-thalamic circuits^62^. Delta-rhythmic neurons, both ChIs and SYNs, had intraburst firing rates in the beta frequency range, indicating a potential spike-related delta-beta coupling mechanism. However, the observations of increased Vm beta power in the absence of spikes demonstrate that beta rhythms also rely on network mechanisms. Beta power enhancement was limited around particular phases of the Vm delta, highlighting the transient nature of beta rhythms^21^. Sustained and exaggerated beta rhythms are a hallmark of Parkinsonian pathology, which likely involves ChIs^39,40^. Given the close link between delta and beta oscillations in healthy animals^4,22,23,25^, delta-rhythmic modulation of beta rhythms in ChIs may be disrupted in the Parkinsonian brain. Future voltage imaging studies in Parkinsonian animal models will provide insights into how the loss of dopamine alters ChIs’ cellular voltage dynamics and contributes to PD circuit pathophysiology.

LFP beta-band power in the motor system is dynamically regulated during locomotion and exhibits overall suppression during motor execution^4,19,26,46^. Here, we found that delta-rhythmic ChI spiking was not associated with any LFP beta power change between resting and locomotion, but rather was accompanied by an increase in high-gamma oscillations, suggesting synchronized striatal neural activity upon ChI activation. In contrast, delta-rhythmic SYN spiking was coupled to decreased LFP beta during locomotion, demonstrating that SYN cellular dynamics contribute more to striatal LFP beta rhythmicity, as LFP beta oscillations were generally suppressed during movement. Furthermore, we also detected significant modulation of LFP beta power by the phase of delta-rhythmic stepping cycles, highlighting that dynamic fluctuations of beta power might support the patterning of movement.

We found 81% of ChIs exhibited delta rhythms, in contrast to 36% of SYNs (primarily SPNs). Here, we cannot delineate whether the delta-rhythmic SYN subset corresponds to a particular type of SPN or to other interneurons. Given the sparsity of ChIs in the striatum (roughly 2%)^43^, it is unlikely that ChIs contributed substantially to the delta-rhythmic SYN population, and the majority of delta-rhythmic striatal neurons are not expected to be ChIs. Furthermore, only the delta-rhythmic subset of SYNs exhibited locomotion-dependent firing rate modulations, suggesting that they are part of a locomotion-sensitive striatal circuit. Non-delta-rhythmic neurons exhibited mainly regularly spaced firing patterns, though we noted some neurons showing theta-burst spiking patterns. Future studies delineating other neuron subtypes could reveal the distinct functions of different striatal cell types in locomotion and movement patterning.

## Methods

### Mouse surgery

All animal experiments were performed in accordance with the National Institute of Health Guide for Laboratory Animals and approved by the Boston University Institutional Animal Care and Use and Biosafety Committees. Same-sex mice from the same litters were generally housed together prior to surgery and single-housed post-surgery. Enrichment in the form of Igloos and running wheels was provided. Animal facilities were maintained around 70 °F and 50% humidity and were kept on a 12 h light/dark cycle.

We used a total of 12 adult mice including five ChAT-Cre mice (three males, two females; ChAT-cre; Tg(Chat-cre)GM24Gsat/Mmucd, MMRRC-017269-UCD, NIH MMRRC) injected with FLEX-SomArchon, three ChAT-Cre mice (two females and one male) injected with syn-SomArchon, and four ChAT-tdT mice (four females; crossed between ChAT-Cre and the tdT mouse line: B6.Cg-*Gt(ROSA)26Sor*^*tm14(CAG-tdTomato)Hze*^/J from The Jackson Laboratory) injected with syn-SomArchon. Mice were 10–22 weeks old at the start of the study. Data from male and female mice were pooled for the analysis. No statistical method was used to predetermine the sample size.

#### Striatum imaging window implantation

Striatal window surgeries were performed similarly to those described previously in refs. ^35, 41, 46, 63, 64^. Custom imaging windows consisted of a stainless steel cannula (OD: 3.17 mm, ID: 2.36 mm, 1 or 2 mm height, AmazonSupply, B004TUE45E) with a circular coverslip (#0, OD: 3 mm, Deckgläser Cover Glasses, Warner Instruments Inc., 64-0726 (CS-3R-0)) adhered to the bottom using a UV curable glue (Norland Products Inc., Norland Optical Adhesive 60, P/N 6001). We glued a virus infusion cannula (26 G, PlasticsOne Inc., C135GS-4/SPC), and an LFP electrode made of stainless steel wire (Diameter: 130 µm, PlasticsOne Inc., 005SW-30S, 7N003736501F) to the side of the imaging window using super glue (Henkel Corp., Loctite 414 and Loctite 713). The LFP electrode protruded from the bottom of the imaging window by about 200 µm.

A craniotomy ~3 mm in diameter was made over the striatum (AP: +0.5, ML: −1.8). A small notch was made on the posterior edge of the craniotomy to accommodate the infusion cannula and LFP recording electrode. The overlying cortex was gently aspirated using the corpus callosum as a landmark. The corpus callosum was then carefully thinned to expose the dorsal striatum. The imaging window was positioned in the craniotomy, and Kwik sil adhesive (World Precision Instruments LLC, KWIK-SIL) was applied around the edges of the imaging window to hold it in place and to prevent any dental cement from touching the brain. Three small screws (J.J. Morris Co., F000CE094) were screwed into the skull to further anchor the imaging window to the skull, and a small ground pin was inserted into the posterior part of the brain near the lambda suture as a ground reference for LFP recordings. Dental cement was then gently applied to affix the imaging window to the exposed skull, and to mount an aluminum headbar posterior to the imaging window.

AAV virus injection occurred either one week prior to window implantation surgery, or through the virus infusion cannula after window implantation surgery. All AAVs were produced by the University of North Carolina Chapel Hill Vector Core. Sequences of the proteins used in this study are available at Genbank at the following accession code: SomArchon, MN091368. Plasmids for the viruses used in this study and their sequences are available at Addgene.org (pAAV-CAG-FLEX-SomArchon, Addgene# 126943; pAAV-syn-SomArchon, Addgene# 126941). AAV-Syn-SomArchon (5.9e12 genome copies (GC)/ml) or AAV-CAG-FLEX-SomArchon (6.3e12 GC/ml) was injected into the dorsal striatum (AP:+0.5, ML:−1.8, DV:−2.2, 1 uL virus). Viral injection occurred at 50–100 nL/min. In mice where AAV was injected during surgery, AAVs were infused with a 10uL syringe (NANOFIL, World Precision Instruments LLC) fitted with a 33 gauge needle (World Precision Instruments LLC, NF33BL) and controlled by a microinfusion pump (World Precision Instruments LLC, UltraMicroPump3–4). In mice where AAV was infused after window implantation, 1 uL of AAV virus was infused through an internal infusion cannula (33 G, PlasticsOne Inc., C315IS-4/SPC) connected to a microinfusion pump (World Precision Instruments LLC, UltraMicroPump3–4), approximately 1 week after the window implantation surgery. The internal infusion cannula was left in place for 10 minutes following injection to facilitate viral spread. Mice were awake and head-fixed throughout the injection period.

All mice were treated with buprenex (0.1 mg/kg) for 48 h following surgery or with sustained release (SR) buprenorphine (3.25 mg/kg) at the beginning of surgery and single-housed to prevent any damage to the headbar or window implant.

### Head-fixed voluntary movement experiments

All voluntary movement experiments were performed while animals were awake. Head-fixed mice were allowed to freely navigate using a treadmill made of a Styrofoam ball as previously described in refs. ^35, 41, 46^, but pinned along the center axis to restrict free movement to forward or backward motion only (no lateral movement). The movement was tracked using two computer mouse sensors positioned roughly ±75 degrees from the center along the equator of the ball. In order to determine the mouse movement speed, the pinned ball was rotated vertically to calibrate sensor displacement. All motion sensor displacement data were acquired at 20 Hz on an Arduino Teensy board and synthesized using a custom Matlab script. The timing of each motion sensor displacement data point was also recorded using the OmniPlex system (PLEXON Inc.) for offline synchronization with optical voltage recordings.

All mice were habituated on the treadmill for at least three days, at least 20 min per day, prior to image acquisition. During optical imaging, mice were imaged while freely navigating the treadmill.

### Local field potential recordings

LFPs were recorded using OmniPlex (PLEXON Inc.) at a 1 kHz sampling rate. To synchronize voltage imaging and LFP recordings during offline data analysis, the OmniPlex system also recorded the TTL pulses that were sent by the sCMOS camera at the onset of each image frame.

### SomArchon voltage imaging

All optical recordings were acquired on a custom widefield fluorescence microscope equipped with a Hamamatsu ORCA Fusion Digital CMOS camera (Hamamatsu Photonics K.K., C14440-20UP), 10x NA0.25 LMPlanFI air objective (Olympus Corp.), 40x NA0.8 LUMPlanFI/IR water immersion objective (Olympus Corp.), 470 nm LED (ThorLabs Inc., M470L3), a 140 mW near-infrared 637 nm laser (Coherent Obis 637-140X), a green filter set (Olympus, OCT49002BX3) with a 470/25 nm bandpass excitation filter, a 495 nm dichroic, and a 525/50 nm bandpass emission filter, and a near-infrared filter set with a 635 nm laser dichroic filter, and a 664 nm long pass emission filter (Olympus, OCT49006BX3). The near-infrared laser illuminated a circular area of ~70 µm in diameter, with a field of view (FOV) size of ~50 µm × 70 µm under the 40x objective lens. Our previous computational modeling study showed that fluorescence crosstalk is negligible when nearby fluorescent neurons were >30 um away laterally, regardless of axial distance^63^. We thus primarily recorded from FOVs with only one or two neurons, to minimize potential fluorescence crosstalk. A mechanical shutter (Newport Corporation, model 76995) was positioned in the laser path to control the timing of illumination over the imaging window.

For each FOV, we first collected the GFP fluorescence of the SomArchon-GFP fusion protein in the green channel (λ_ex_ = 470 nm) at 1024 × 1024 pixels with 2 × 2 binning to capture cell morphology. SomArchon optical voltage recordings were then performed in the near-infrared channel (λ_ex_ = 637 nm) with 2 × 2 binning. Optical recordings were acquired at ~833 Hz with HCImage Live (Hamamatsu Photonics K.K.). HC Image Live data were stored as DCAM image files (DCIMG) and analyzed offline in Fiji/ImageJ and MATLAB (Mathworks Inc.). To synchronize optical recordings with LFP recordings, the camera sent out a TTL pulse to the OmniPlex system (PLEXON Inc.) at the onset of imaging and after each acquired frame. During each recording, we first performed a test trial to ensure spiking activity was present in the putative neuron soma before running the full recording protocol of up to ten trials, 12 s per trial with an inter-trial interval of at least 30 s in duration to reduce photobleaching.

For each recorded neuron, SomArchon fluorescence traces were first visually inspected to ensure a lack of significant image motion and reasonable signal-to-noise level based on spike appearance before further analysis. Neurons that passed this visual inspection stage were further analyzed, resulting in 27 ChIs (mean ± standard deviation: 4.5 ± 2.1 ChIs per mouse, *n* = 6 mice) and 25 SYNs (3.6 ± 3.5 SYNs per mouse, *n* = 7 mice).

### Voltage imaging data motion correction and ROI identification

Motion correction was performed with a custom Matlab script. SomArchon fluorescence images were first motion corrected using a pairwise rigid motion correction algorithm as described previously^41,63,64^. Briefly, the displacement of each image was computed by identifying the max cross-correlation coefficient between each image and the reference image. Our recordings consisted of multiple 12-s-long trials. Each video file corresponding to one trial was first concatenated into a multi-trial data matrix, after which the motion correction algorithm was applied. Since the laser illumination area was about 70 µm in diameter, a rectangular window large enough to cover the entire neuron across all frames was selected manually for motion correction. The window selection was chosen to avoid dark regions of the image and to include regions that had distinguishable cell-like contrasts to facilitate comparison with a reference image. Each trial was first motion corrected individually. We then corrected image shifts across trials by referencing all trials to the first trial. The motion-corrected image sequences were then used for subsequent manual ROI neuron identification using the drawPolygon function in Matlab. SomArchon fluorescence traces for each ROI were then extracted from the motion-corrected image sequences. The optically-recorded voltage traces for each ROI were generated from the motion-corrected image sequences and were then used for analyses. Due to photobleaching, the quality of SomArchon recording decreases over time. We, therefore, excluded trials (10.95%) where the spike-to-baseline ratio (SBR, see below) dropped below an SBR average of 5. Further, due to the sensitivity of Vm traces to image motion, for Vm analysis, we excluded time periods (26.26%) for each recording where there was image motion of >0.065 µm/ms calculated as the combined rectified X-Y derivative of the image motion.

### SomArchon fluorescence detrending and spike detection

All optically-recorded SomArchon traces reported in the manuscript were corrected for photobleaching by subtracting the smoothed trace using the Matlab function fastsmooth (https://www.mathworks.com/matlabcentral/fileexchange/19998-fast-smoothing-function). Spike detection was performed similarly to that described previously in ref. ^63^. To estimate baseline fluorescence, we first averaged the fluorescence trace using a moving window of ±100 frames to obtain the “Smoothed Trace” (ST). We then removed potential spike contributions to the baseline by replacing fluorescence values above the ST with the corresponding ST values, which resulted in a spike-removed trace including only the subthreshold baseline fluctuation. To identify spikes, SomArchon fluorescence traces were high-pass filtered (>120 Hz), and spikes were identified as fluorescence increases greater than 4 standard deviations above baseline subthreshold fluctuations.

### Signal-to-baseline ratio (SBR) calculation

To calculate SBR^63^, we first determined the spike amplitude by calculating the difference between the lowest and peak spike fluorescence value within three data points prior to the spike. We then divided the spike amplitude by the standard deviation of the Vm across the entire recording duration.

### Subthreshold membrane voltage (Vm) traces

To obtain subthreshold membrane voltage (Vm) traces, we removed three data points around the peak of each detected spike from the non-filtered SomArchon dF/F trace and interpolated the missing data points using the surrounding data points (*n* = ±3 points).

### SomArchon voltage imaging, LFP, and animal locomotion data alignment

Voltage imaging data, LFP data, and animal locomotion data were aligned to the camera start trigger (first frame) of each trial and interpolated to a frame rate of 1 kHz to match that of the LFP recordings. Subsequent analyses were performed on these aligned and interpolated data.

### Inter-spike interval (ISI) calculation

Inter-spike intervals were calculated as the time between identified spikes in milliseconds. For a given spike, we computed the time difference between the previous spike (ISI n) and the following spike (ISI *n* + 1). The two-dimensional space of ISI n and ISI *n* + 1 represented the so-called ISI return map. Plotting the ISI return map, on a logarithmic scale, helped to visualize the bursty and delta-rhythmic spiking patterns of many neurons, particularly striatal ChI neurons.

### Neuron classification based on ISI profile

We observed neurons with distinct ISI structures. Particularly, a subset of neurons exhibited strong delta-rhythmic (2–3 Hz) spiking patterns. Other neurons had more regular spiking patterns. We also observed other spiking patterns, including theta-bursting neurons (5–8 Hz) and fast-spiking neurons, but due to their low numbers, they were not analyzed separately. All neurons that did not exhibit a clear delta-rhythmic spiking component constituted the non-delta group. To capture the different types of single neuron ISI curves systematically, we computed an ISI ratio (A − B)/(A + B) defined as the number of spikes with ISIs of 300–700 ms (1.3–3.3 Hz) being A and the number of spikes with ISIs of 80–200 ms (5–12.5 Hz) being B. This ratio captured neurons with prominent ISI delta peaks. To categorize each neuron as delta-rhythmic versus non-delta, we first manually inspected each neuron’s ISI return map, and determined that delta rhythmic ISIs could be well separated from other neurons using the ISI ratio 0. We thus defined neurons as delta-rhythmic which had an ISI ratio of at least 0 or more. Most delta-rhythmic neurons spiked several times per cycle, however, a few neurons only had one spike per cycle.

### Spike phase-locking computation

To quantify how consistent spikes occurred relative to the oscillation phase, we first calculated the phase-locking value (PLV^65^) defined as:

1 $$\,{{{{{{\mathrm{PLV}}}}}}}\left(f\right)=\left| \frac{1}{N} \right|\mathop{\sum}\limits_{N}{e}^{i{{\varnothing }}(f,n)}$$

where *f* is the frequency and *N* is the total number of spikes. The phase ϕ was obtained from the complex wavelet spectrum.

Since PLV is not independent of the number of spikes considered and tends to inflate with low numbers of spikes, we only included neurons that had at least ten spikes for spike-PLV analysis. Further, we adjusted the PLV value using the following equation^66^ to account for any potential differences in the number of spikes between groups of neurons, which we term here as unbiased phase locking value (PLVu^2^):

2 $${{{{{{\mathrm{PLV}}}}}}}{u}^{2}\left(f\right)=\frac{1}{N-1}({{{{{{{\mathrm{PLV}}}}}}}\left(f\right)}^{2}{\times N}-1)$$

where *N* is the number of spike occurrences and *f* is frequency. The unbiased PLVu^2^ corresponds, at a larger *N*, to the default PLV^2^.

### Spike-triggered Vm and LFP spectrograms

All spectrograms were calculated with the FieldTrip toolbox (https://www.fieldtriptoolbox.org/) for Matlab using the wavelet method (Morlet wavelets). Spectrograms for each neuron were aligned to each spike and the time window of 250 ms before and after each spike was averaged to create a spike-triggered spectrogram per neuron (as in Fig. 3c). Population spectrograms shown in Figs. 3d–f, h–j, 5e–g and Supplementary Figs. 5, 8 were created by averaging across the spike-triggered (or delta cycle peak-triggered) spectrograms for delta-rhythmic neurons, non-delta rhythmic neurons, and SYN or ChI populations, respectively. For Supplementary Fig. 8 spectrograms were aligned only to spikes that occurred in rest or movement periods respectively, and these spectrograms were subtracted to create those in Fig. 5e–g. Spectrograms were normalized to the averaged pre-spike power between −200 to −100 ms before the trigger. The spectrogram in Fig. 3c was created using the wavelet method on the corresponding spike-triggered Vm trace shown in Fig. 3b. Figure 3g, k display the power ratio between the time point of spike occurrence and the pre-spike period (−200 to −100 ms before the spike) at beta frequency (20–40 Hz). Only neurons with a minimum number of 20 triggered windows were included in the analysis.

### Spike raster plot

For the example neuron in Fig. 4e, the spike timing was plotted per trial aligned to movement onset. The average spike rate was then calculated across all onsets for the neuron.

### Autocorrelograms and FWHM calculation

For Fig. 2e–g, we computed the spike train and Vm autocorrelograms using the xcorr matlab function. Shown are time lags from +3 to +2500 ms, as at time 0 the plot is dominated by the perfect autocorrelation peak (correlation with itself). To compute the instantaneous frequency distribution (Fig. 2f, g), we filtered the Vm signals in the broad delta frequency range (1–6 Hz) to capture most of the frequency variation. We then applied a Hilbert Transform to obtain the analytical signal from which we derived the instantaneous frequency (IF). To obtain IF estimates, we unwrapped and smoothed the instantaneous phases with a savitzky-golay filter with polyorder = 2 and frame size = 201 ms and differentiation order = 1. For the peak frequency estimate in Fig. 2h, i, for each neuron, we selected the frequency with the maximum probability of occurrence. To estimate the full-width-at-half-maximum (FWHM) for each neuron, we quantified the lowest and highest frequency that fulfilled the condition of being half the maximum probability of the instantaneous frequency distribution.

### Definition of movement periods and transition points

To identify movement bouts, animals’ movement data was first smoothed using a 1.5 Hz low pass Butterworth filter to define the transitions more robustly. We defined low-speed (“rest”) periods as intervals where the speed was below ≤5 cm/s and high-speed (“movement”) periods as intervals where the speed was above ≥5 cm/s. A movement transition was defined as the point at which a pre-defined rest period and movement period intersect, where a rest-to-movement transition (or “onset”) was identified by a movement period directly following a rest period, and vice versa for a movement-to-rest transition (or “offset”) where a rest period directly followed a movement period. In Fig. 4b, c and Supplementary Fig. 6, the firing rates during these rest and movement periods were compared. For each neuron, we calculated the average spike rate per second in rest periods (total spikes/number of rest frames x1000) and the average spike rate per second in movement periods across trials. The difference between the average spike rates in rest and movement periods was used as the metric. To determine if a neuron was responsive to sustained periods of movement, we built a basal distribution of the differences using shuffling. We randomly chose the same number of frames as the total rest frames in each trial and calculated the spike rate across these randomly selected frames and finally took an average across trials for every neuron. This was repeated to calculate the average spike rate in random movement periods as well. The difference between these random spike rates was calculated and this procedure was repeated 1000 times. If the observed difference for a neuron was beyond the 97.5th percentile of the distribution, then the neuron was classified to exhibit a significantly increased response during movement. On the other hand, if the observed difference was less than the 2.5th percentile of the distribution, the neuron was classified to exhibit a significantly decreased response during movement.

### Movement-triggered spike rate

For each neuron, the average spike rate across one-second time windows (500 ms before and after each transition) was calculated in 100 ms intervals and averaged across all onset or offset transitions per neuron, respectively. To determine the statistical significance of these movement-triggered spike rates compared to chance, shuffling was used. For each neuron, the same number of points as the number of onset transition points were randomly selected. One-second time windows (500 ms before and after each transition) around these random transition points were concatenated across all transitions. The windows across neurons were concatenated and an average across these windows was calculated. Then, the average spike rate was calculated as the moving mean over a sliding window of 100 ms. This shuffling was done 1000 times to build a basal distribution of average spike rates. The actual metric from observation was also calculated in the same manner using the identified onset transition points. If the calculated movement-triggered spike rate exceeded the 97.5th percentile or was lower than the 2.5th percentile (equivalent to being beyond 2 standard deviations), then the spike rate was deemed to be significant and *p* value was calculated. Significance was determined if a spike rate exceeded ±2 standard deviations above or below the shuffled chance value.

### Animal limb motion tracking

To correlate the animal’s limb positions with treadmill speed, we performed simultaneous video recordings of the animal’s limbs while recording treadmill movement speed, as described earlier (**Head-fixed voluntary movement experiments**). Video recordings of the limbs were performed with two Logitech C910 webcams, with each camera capturing the hindlimb and the front limb on each side of the body at 120 Hz. Videos were collected with the open-source software OBS studio (https://obsproject.com/), and offline trimmed and cropped using the FFmpeg software (https://ffmpeg.org/). To synchronize video recordings and treadmill speed recordings, an LED light was activated at the start of each recording via TTL pulses generated by the OmniPlex system (PLEXON). As was described for voltage imaging experiments, the OmniPlex system also recorded the time stamps from each movement sensor used to monitor treadmill speed. We then identified the video frame where the LED light was first detected and used that frame to align limb videos to treadmill speed recordings.

Limb videos were offline analyzed using DeepLabCut^67^. Specifically, we first used DeepLabCut to identify 20 representative frames per training video using the built-in k-means clustering algorithm. We manually marked the hindlimb and the front limb positions on these representative frames as ground truth frames. We then trained a deep neural network using ground truth frames from 13 videos from three mice recorded. Ground truth frames were iteratively improved by refining the limb positions on frames that were identified as outliers by DeepLabCut during the training process. The tracking performance of the final DeepLabCut deep neural network was evaluated by manual visual inspection and quantified by the likelihood value for each frame. The mean average Euclidean error (MAE, proportional to the average root mean square error) was calculated between the predicted and actual user-labeled positions to evaluate the performance of the network. The MAE for the training set (95% of the manually labeled frames) was 2.36 pixels, and for the test set (5% of the manually labeled frames) was 5.21 for all frames or 4.52 for frames with a likelihood greater than 0.6, compared to the average limb area of 1353 pixels (averaged over 48 frames identified in ImageJ).

The limb coordinates (x, y) were used to calculate the relative position of each limb as the resultant vector length. Once treadmill speed traces were aligned with limb positions, coherence between the two signals (treadmill speed, left hindlimb) was computed using the coherence function from the fieldtrip toolbox (http://fieldtriptoolbox.org). Periods of limb locomotion were defined as when the amplitude envelope of the left hindlimb signal derivative exceeded >0.1. To obtain a randomized distribution, we first flipped the trial time of the treadmill speed signals, which removes the temporal correspondence between the treadmill speed trace and the left hindlimb position. We then shuffled the labels of the true and randomized data and calculated the coherence values to obtain a shuffled null distribution, which was then used to assess the likelihood of the true coherence value relative to the shuffled null distribution (*p* < 0.05, Fig. 6d). Average left and right hindlimb positions were then plotted relative to peaks in the delta-filtered treadmill speed traces (Fig. 6e).

### Treadmill speed delta phase analysis

In the unprocessed treadmill speed traces, rhythmic fluctuations can be observed during animal movement (e.g., see Fig. 4a). Spectral analysis showed that the rhythmicity has a peak in the delta range with a dominant peak around 2–3 Hz. To compute the phase-locking of striatal neuron spiking to treadmill speed, as well as for analysis of treadmill delta-peak triggered Vm power, we first extracted the instantaneous phase of the delta-rhythmic treadmill speed signals. If not otherwise mentioned, we bandpass filtered the treadmill speed data between 1–4 Hz with a Butterworth filter. To obtain the instantaneous phase, we applied the Hilbert Transform providing the analytical signal from which the phase could be derived. Phase locking between spiking (Fig. 7a, b, d) or LFP (Fig. 7e) and specific frequencies of treadmill speed were then conducted as described in **Spike phase locking strength calculation** above. In Fig. 7a, b, data were separated between movement and resting periods, while in Fig. 7d, spike phase-locking analysis was performed to delta-filtered treadmill speed at 1–2.6 Hz or 2.8–6 Hz.

Polar histograms in Fig. 6h, j were created by plotting the preferred spiking phase of each neuron relative to the delta-filtered treadmill speed signal. Polar histograms in Fig. 7g, h depict the preferred phase of beta (20–40 Hz) or high gamma (70–100 Hz) power relative to the delta-frequency filtered treadmill speed signals. Each dot represents an LFP recording session and the shaded regions across all plots represent the preferred phase (the phase with the highest mean power). The Omnibus test for non-uniformity was performed to test whether the distribution of preferred phases differed from uniformity as expected from a random process.

The LFP spectrogram in Fig. 7f was created similarly as described in **Spike-triggered Vm and LFP spectrograms** above, but instead triggered to the peaks of delta-filtered treadmill speed traces. Power was normalized to the mean power across the delta-peak triggered window, averaged across all delta peaks and recording sessions. Similarly, the spectrogram in Supplementary Fig. 10 was triggered to left hind-limb (LHL) cycle trough and power was normalized to the mean power across the window and averaged across LHL cycles and recordings.

### Histology

Mice were transcardially perfused with PBS followed by 4% paraformaldehyde. The brain was gently extracted from the skull and post-fixed in 4% paraformaldehyde for 1–4 h at room temperature or overnight at 4 ^o^C. Fixed brains were transferred to a 1% polyvinylpyrrolidone (PVP-40), 30% sucrose, and 30% ethylene glycol PBS-based solution and stored at 4 ^o^C. Before slicing, brains were moved to 30% sucrose-PBS solution and rotated 24–48 h at 4 ^o^C to allow the cryoprotectant solution to diffuse out. Brains were sliced (coronally) to 50 µm thickness using a freezing microtome. Staining of SPNs was performed with primary antibody rabbit anti-DARPP-32 (1:500, Abcam ab40801 [EP720Y]), followed by the secondary antibody AlexaFluor568 (1:500, goat anti-rabbit IgG, Invitrogen A11011). All antibodies were used according to the protocols that have been validated by suppliers. Slice imaging was performed using an Olympus FV3000 scanning confocal microscope equipped with 405, 488, 561, and 640 nm solid-state diode lasers and a 20×, NA = 0.45 air objective lens (LUCPLFN20X; Olympus), controlled by Fluoview FV31-SW software. Acquired images were analyzed in Fiji/ImageJ.

### Statistics and reproducibility

Unless otherwise specified, all between-group statistics shown in violin plots (Figs. 1m–p,  2c, d, l, m, p, q,  3a, g, k,  4h,  5d, h,  7c) were conducted using a two-sample independent student *t*-test. For within-group statistics (Figs. 3g, k,  4h,  5d, h,  7c), a one-sample student *t*-test was used. A *p* value threshold of *p* ≤ 0.05 was used to determine significance. Figures 3g, k, 5h are compared to the pre-spike condition −200 to −100 ms before the spike. Statistics in Fig. 4b–d, h are between the rest and movement periods or the pre- and post-transition periods, respectively. Neurons that did not meet the criteria for sufficient numbers of spikes, low vs. high movement periods, or movement transitions were excluded from relevant statistical analyses.

### Reporting summary

Further information on research design is available in the Nature Portfolio Reporting Summary linked to this article.

## Supplementary information

Author Meta Data

Supplementary Information

Description of Additional Supplementary Files

Supplementary Software

Reporting Summary

## Data Availability

The source data for all relevant statistics and example recordings are provided in the Source Data file, and are also available at the GitHub repository: https://github.com/HanLabBU/Shroff-Lowet-Nature-Communication-2023 (https://zenodo.org/record/7805663#.ZC8kEXYpD30). The experimental raw data that support the findings of this study are available from the lead contact upon request. Source data are provided with this paper.

## Source data

Source Data

## Code availability

Codes used for data analysis is available at the Github repository: https://github.com/HanLabBU/Shroff-Lowet-Nature-Communication-2023 (https://zenodo.org/record/7805663#.ZC8kEXYpD30) and in Supplementary Software 1. Any additional information required to reanalyze the data reported in this paper is available from the lead contact upon request.
