# Supplementary material for: Striatal cholinergic interneuron membrane voltage tracks locomotor rhythms in mice
Source: Nat Commun. 2023 Jun 26;14:3802. doi: 10.1038/s41467-023-39497-z (PMC10293266; doi:10.1038/s41467-023-39497-z)
Supplement: Supplementary file 2 — Supplementary Information [file 41467_2023_39497_MOESM2_ESM.pdf]

Striatal cholinergic interneuron membrane voltage tracks locomotor rhythms in mice

Supplementary Figures and Legends

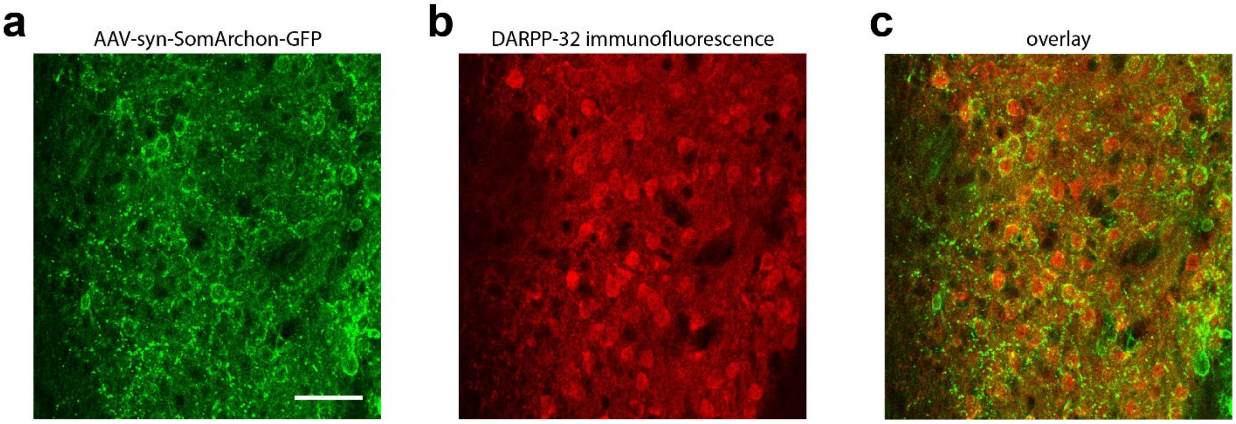

Supplementary Figure 1. Neurons labelled with AAV-syn-SomArchon are mainly medium spiny projecting neurons (SPNs). *(**a**). An example dorsal striatal brain slice, showing GFP fluorescence (λ=488nm) in neurons expressing SomArchon-GFP fusion proteins transduced with AAV-syn-SomArchon in a C57BL6 mouse. (**b**). Immunofluorescence of DARPP-32 (AlexaFluor568, λ=568nm), an SPN-specific neuronal marker, in the same brain slice as **a**. (**b**). Overlay of **a** and **b**. Scale bar is 50μm.*

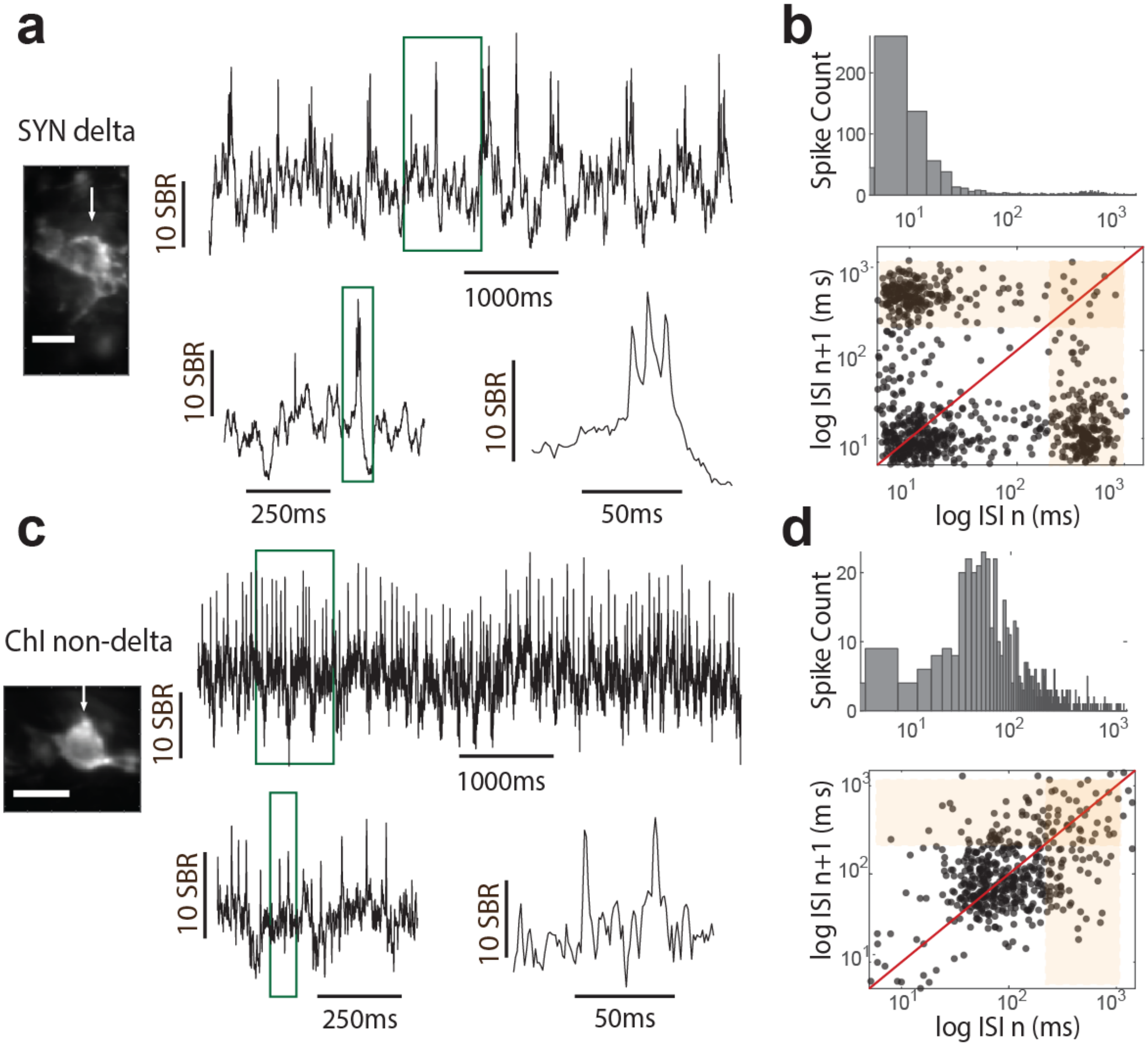

Supplementary Figure 2. An example delta rhythmic SYN and non-delta rhythmic ChI. ***(a)***. *An example SomArchon fluorescence recording from a delta-rhythmic SYN (sampling rate at 833Hz). Left, SomArchon fluorescence image, with the example neuron indicated by an arrow. Right, 6 seconds long SomArchon fluorescence trace is shown for the example SYN, with sequential zoom in of the periods indicated by the dashed green boxes.* ***(b).*** *Top, the inter-spike interval (ISI) distribution of the example SYN shown in* ***a****. Bottom, the return map, plotted as ISI (n) vs ISI (n+1), for the example SYN shown in* ***a****. Orange shading indicates the delta rhythmic band. The X-and Y-axis are in logarithmic scale. The bin size in the top ISI distribution is fixed at 5ms.* ***(c)****. Same as* ***a****, but for an example non-rhythmic ChI.* ***(d)****. Same as* ***b****, but for the example neuron shown in* ***c****. Scale bars are 15µm. Source data are provided as a Source Data file.*

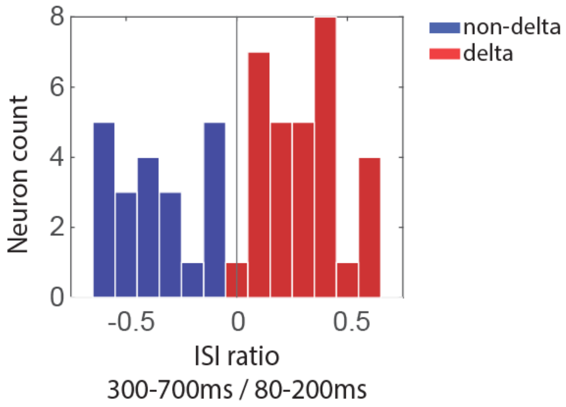

Supplementary Figure 3. Categorization of delta-rhythmic and non-delta neurons by inter-spike interval ratio. *The delta-rhythmicity in the inter-spike intervals (ISI) was quantified for each neuron as the ratio (A-B)/(A+B) between the average ISI probability over the 300-700ms range (A) and the averaged ISI probability quantified over the 80-200ms range (B). An ISI ratio threshold of 0 is used to determine whether a neuron was classified as non-delta (blue, ND, n=21) or delta-rhythmic (red, D, n=31). Source data are provided as a Source Data file.*

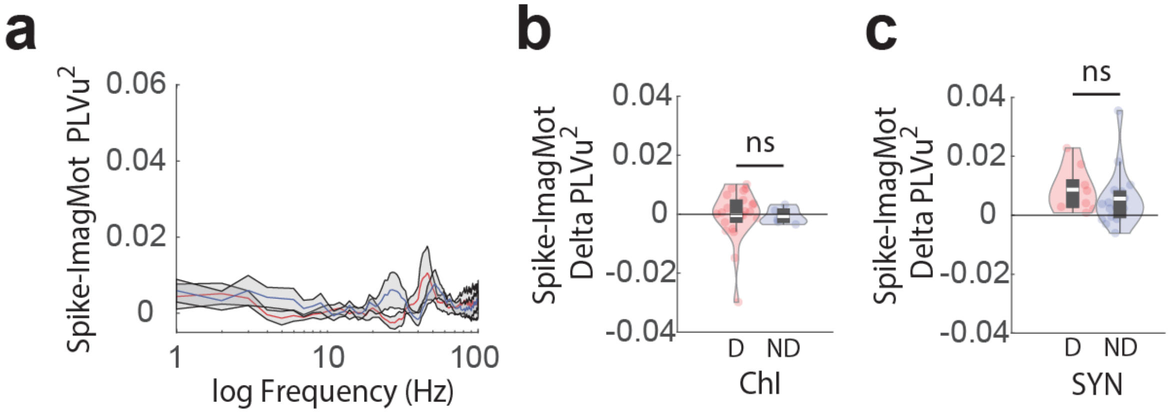

Supplementary Figure 4. The relationship between spikes and image motion is weak and not different between neuron groups. ***(a)*** *Estimation of squared phase-locking value (PLVu^2) of spikes to image motion displacement for delta-rhythmic (red) or non-delta neurons (blue). Image motion displacement calculated as frame shift during motion correction ($\sqrt{X^2 + Y^2}$ image pixel displacement), corresponding to the spatial shift of neurons within an FOV, inherent for awake, behaving conditions with intrinsic biological motion e.g. breathing, heartbeat, movement. Instrument noise (vibration of cooling mechanical fan for sCMOS camera) was also observed around 80Hz. Solid lines represent the population means of delta-rhythmic (red, n=31) and non-delta (blue, n=21) neurons. Phase-locking of image motion with delta-rhythmic vs. non-delta neuron spiking at delta frequency was not significantly different.* ***(b)*** *Quantification of the PLVu^2 for spikes to image motion displacement at delta frequency (1-4Hz) between delta-rhythmic ChIs (D, red) and non-delta ChIs (ND, blue, independent t-test, ChI, D vs ND: p=0.94, n = 27, df=25).* ***(c)*** *Same as* ***b****, but for SYNs (independent t-test, SYN, D vs ND: p=0.43, n = 25, df=23). Shaded regions around lines represent standard error of mean. Violin plots represent the data kernel density with a box-and-whisker plot (box: interquartile range, whiskers: 1.5x interquartile range, white line: mean). All statistical tests are two-sided. Source data are provided as a Source Data file.*

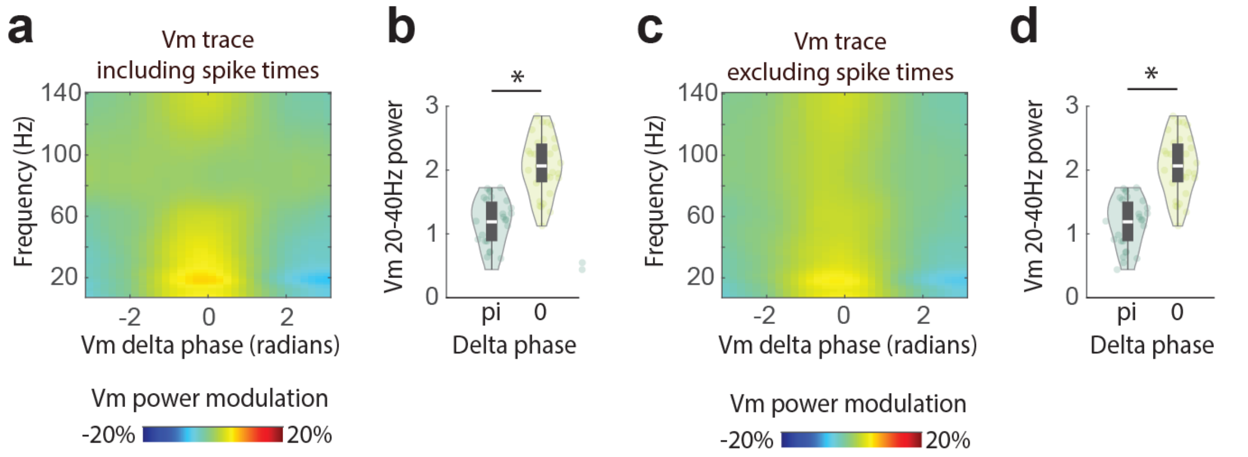

Supplementary Figure 5. Vm delta-phase dependent beta power is independent of spiking activity. ***(a).*** *Normalized Vm spectrum power aligned to Vm delta-peak (relative to pre-peak period of -200 to -100ms) in delta-rhythmic neurons (including both ChIs and SYNs, n = 31) across all delta cycles and* ***(c)*** *only during delta cycles without any spikes.* ***(b).*** *Quantification of Vm beta-band power (20-40Hz) at Vm delta-peak (phase=0°) versus delta trough (phase=180°/pi). Vm beta power at delta peaks is significantly higher than that at delta troughs (paired t-test, n = 31, df=30, with spikes: p=1.15e*$^{-15}$*).* ***(c).*** *Same as* ***a****, but only averaged across delta cycles without any spikes.* ***(d).*** *Same as* ***b****, but for delta cycles without spikes. Vm beta power at delta peaks is significantly higher than that at delta troughs (paired t-test, n = 31, df=30, without spikes: p=1.44e*$^{-15}$*). Overall, Vm beta power was very similarly modulated by Vm delta phase regardless of spikes occurrence, suggesting that spiking is not required for Vm beta power modulation. Quantifications are visualized as violin plots with the outer shape representing the data kernel density and a box-and-whisker plot (box: interquartile range, whiskers: 1.5x interquartile range, white line: mean). All statistical tests are two-sided. All statistical tests are two-sided. Source data are provided as a Source Data file.*

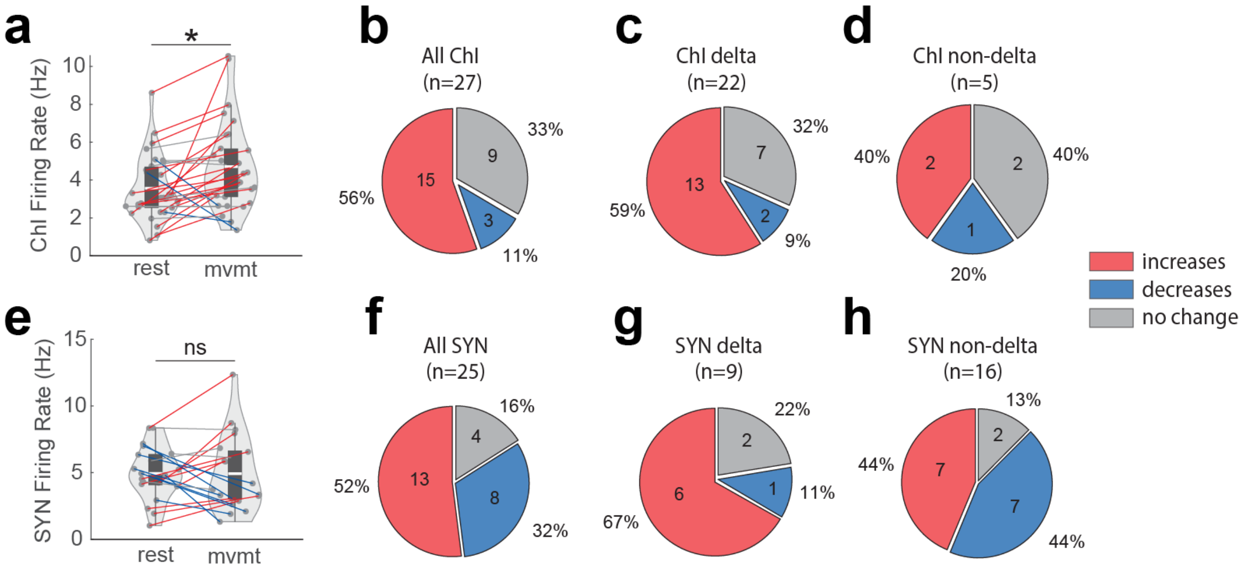

Supplementary Figure 6. The ChI population, but not SYN population, increased firing rate during movement compared to rest. ***(a)***. *Mean firing rates of ChIs during rest vs. movement (mvmt). Red lines mark neurons with >10% firing rate increase during movement compared to rest. Blue lines mark neurons with <10% decreased firing rates, and gray lines mark neurons that are unchanged (<10% modulation in either direction). ChIs as a population significantly increased their firing rate during movement (paired t-test, p=0.0042, n = 27, df =26).* ***(b-d)***. *Pie chart visualization of the fraction of ChIs exhibiting >10% increase (red) in firing rate during movement compared to rest (movement-rest)/(movement+rest), > 10% decrease (blue), or no change (<10% modulation).* ***(b)*** *Pie chart including all ChIs,* ***(c)*** *delta-rhythmic ChIs only, and* ***(d)*** *non-delta ChIs only.* ***(e)***. *Same as* ***a***, *but for SYNs. SYNs, as a population did not change their firing rate (paired t-test; p=0.091, n = 25, df =24).* ***(f-h)*** *Same as* ***b-d***, *but for SYNs***.** *Quantifications are visualized as violin plots with the outer shape representing the data kernel density and box-and-whisker plot (box: interquartile range, whiskers: 1.5x interquartile range, white line: mean). All statistical tests are two-sided. Source data are provided as a Source Data file.*

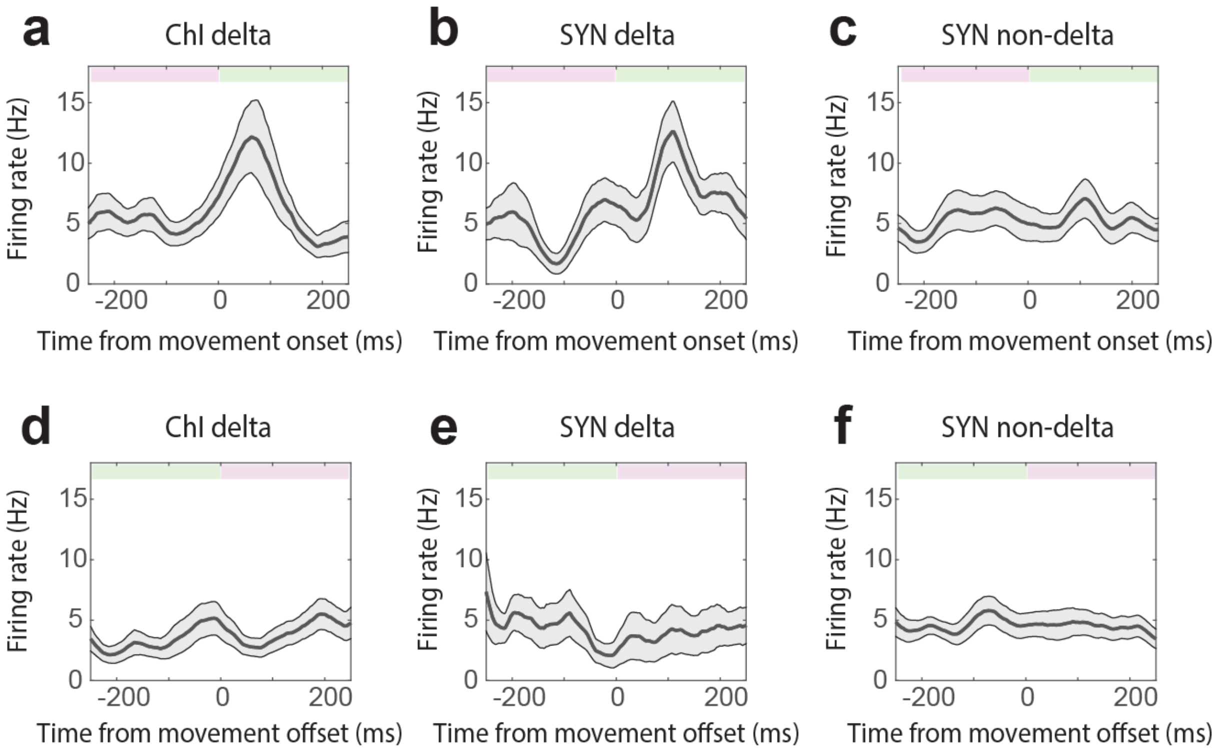

Supplementary Figure 7. ChI and SYN delta-rhythmic neurons, but not non-delta neurons, are differentially responsive to movement onset. ***(a).*** *Delta-rhythmic ChI population firing rate at movement onset (n=21).* ***(b).*** *Delta-rhythmic SYN population firing rate at movement onset (n=9).* ***(c).*** *Non-delta SYN population firing rate at movement onset (n=16).* ***(d).*** *Delta-rhythmic ChI population firing rate at movement offset (n=21).* ***(e).*** *Delta-rhythmic SYN population firing rate at movement offset (n = 9).* ***(f).*** *Non-delta SYN population firing rate at movement offset (n = 16). All shaded regions around lines represent standard error of mean.*

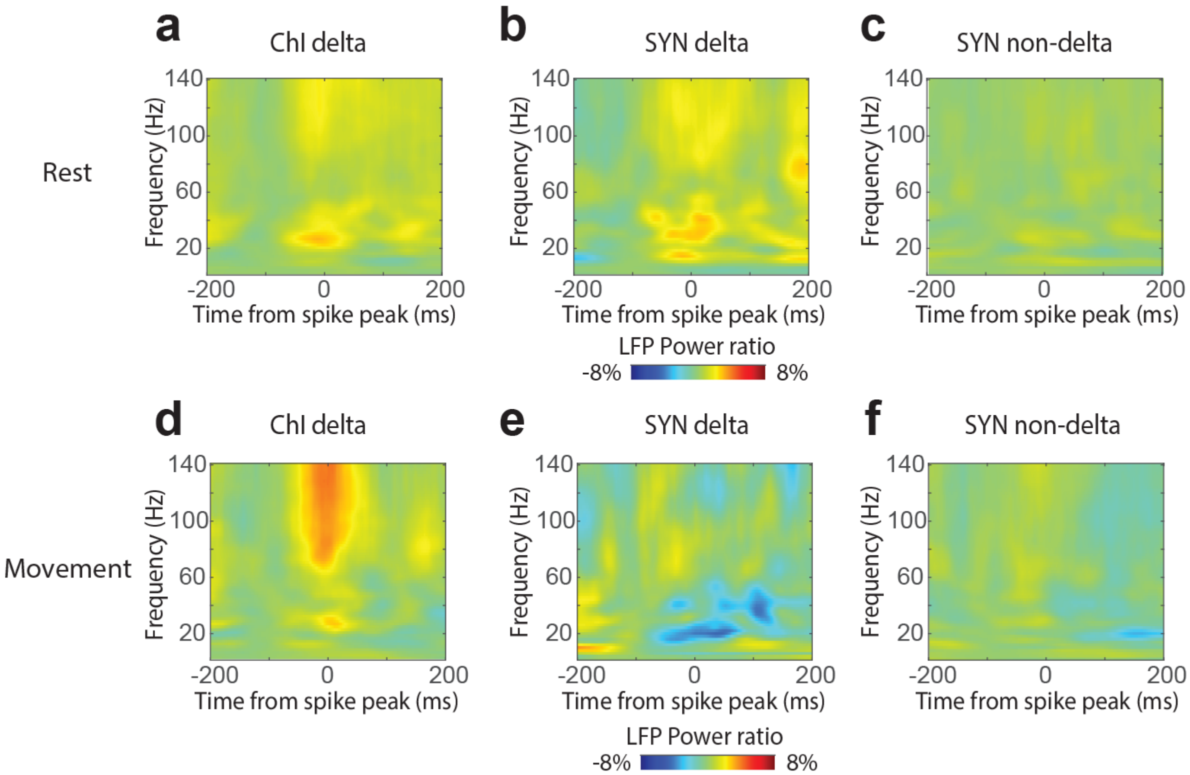

Supplementary Figure 8. Spike-aligned LFP power during rest versus movement. ***(a-c).*** *Population spike-aligned LFP spectral power during rest for* ***(a)*** *delta-rhythmic ChIs (n = 21),* ***(b)*** *delta-rhythmic SYNs (n = 9) or* ***(c)*** *non-delta rhythmic SYNs (n = 16).* ***(d-f)*** *Population spike-aligned LFP spectral power during locomotion for* ***(d)*** *delta-rhythmic ChIs (n = 21),* ***(e)*** *delta-rhythmic SYNs (n = 9) or* ***(f)*** *non-delta rhythmic SYNs (n = 16). LFP spectral power was normalized to the pre-spike period (-200 to -100ms).*

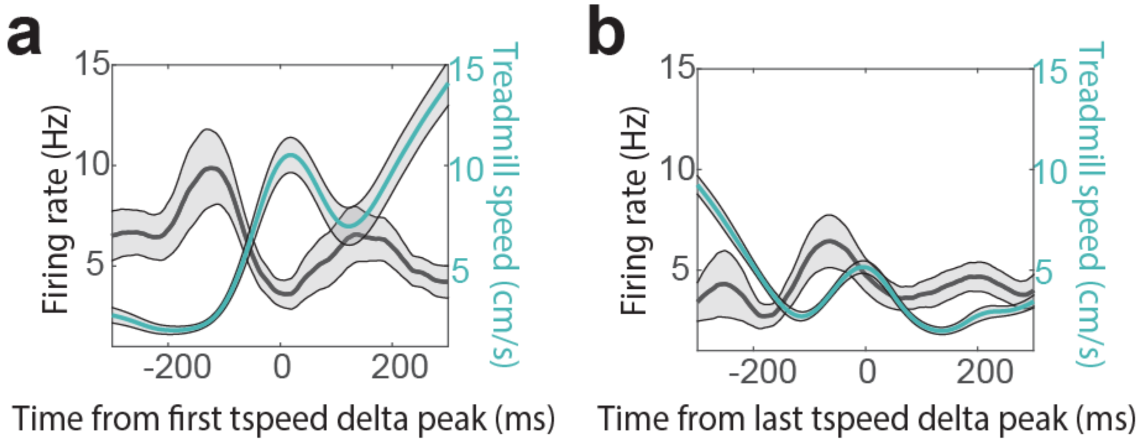

Supplementary Figure 9. Firing rate increases before the first delta cycle peak at locomotion onset and before the last delta cycle peak at locomotion offset. ***(a).*** *Delta-rhythmic neuronal population firing rate aligned to the peak of the first movement-speed delta cycle at locomotion onset (n = 31). Green-blue line represents the averaged treadmill speed.* ***(b).*** *Same as* ***a****, but aligned to the peak of the last movement-speed delta cycle at locomotion offset. All shaded regions around lines represent standard error of mean.*

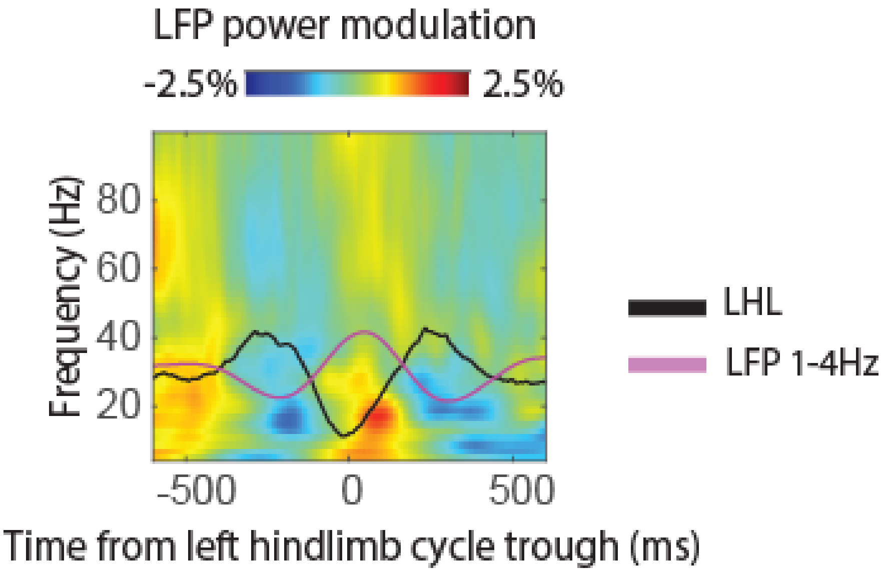

Supplementary Figure 10. LFP delta rhythmicity and beta power is coordinated with animal stepping cycle.
Population LFP spectral power aligned to the trough of animal left hindlimb (LHL) stepping cycle (black), and overlaid with LFP delta cycle (purple).
