# Supplementary material for: Striatal cholinergic interneuron membrane voltage tracks locomotor rhythms in mice
Source: Nat Commun. 2023 Jun 26;14:3802. doi: 10.1038/s41467-023-39497-z (PMC10293266; doi:10.1038/s41467-023-39497-z)
Supplement: Supplementary file 3 — Description of Additional Supplementary Files [file 41467_2023_39497_MOESM3_ESM.docx]

**Description of Additional Supplementary Files**

**Supplementary Software 1:**

**Description:**

All offline analyses were performed with MATLAB (2019b&2020a, Mathworks Inc.). A small dataset has been provided to demo the code.

1. Detailed system requirements of Matlab can be obtained from Mathwork Inc.
2. Installation guide can be obtained from Mathwork Inc.
3. Instructions for use were provided as comments in the code.
