# Supplementary material for: Striatal cholinergic interneuron membrane voltage tracks locomotor rhythms in mice
Source: Nat Commun. 2023 Jun 26;14:3802. doi: 10.1038/s41467-023-39497-z (PMC10293266; doi:10.1038/s41467-023-39497-z)
Supplement: Supplementary file 5 — Reporting Summary [file 41467_2023_39497_MOESM5_ESM.pdf]

nature portfolio

Corresponding author(s): Xue Han

Last updated by author(s): Apr 6, 2023

Reporting Summary

Nature Portfolio wishes to improve the reproducibility of the work that we publish. This form provides structure for consistency and transparency in reporting. For further information on Nature Portfolio policies, see our Editorial Policies and the Editorial Policy Checklist.

Statistics

For all statistical analyses, confirm that the following items are present in the figure legend, table legend, main text, or Methods section.

n/a	Confirmed	
☐	☒	The exact sample size (n) for each experimental group/condition, given as a discrete number and unit of measurement
☐	☒	A statement on whether measurements were taken from distinct samples or whether the same sample was measured repeatedly
☐	☒	The statistical test(s) used AND whether they are one- or two-sided<br>*Only common tests should be described solely by name; describe more complex techniques in the Methods section.*
☐	☒	A description of all covariates tested
☐	☒	A description of any assumptions or corrections, such as tests of normality and adjustment for multiple comparisons
☐	☒	A full description of the statistical parameters including central tendency (e.g. means) or other basic estimates (e.g. regression coefficient) AND variation (e.g. standard deviation) or associated estimates of uncertainty (e.g. confidence intervals)
☐	☒	For null hypothesis testing, the test statistic (e.g. F, t, r) with confidence intervals, effect sizes, degrees of freedom and P value noted<br>*Give P values as exact values whenever suitable.*
☒	☐	For Bayesian analysis, information on the choice of priors and Markov chain Monte Carlo settings
☒	☐	For hierarchical and complex designs, identification of the appropriate level for tests and full reporting of outcomes
☒	☐	Estimates of effect sizes (e.g. Cohen's d, Pearson's r), indicating how they were calculated

Our web collection on statistics for biologists contains articles on many of the points above.

Software and code

Policy information about availability of computer code

Data collection	Data were recorded using HCImage Live, FluoView software, OmniPlex system, OBS studio, FFmpeg software, and MATLAB.
Data analysis	Data were analyzed offline using OmniPlex software, Fiji/ImageJ, DeepLabCut, and MATLAB 2019. Computer code supporting the analyses performed in this study is available https://github.com/HanLabBU/Shroff-Lowet-Nature-Communication-2023

For manuscripts utilizing custom algorithms or software that are central to the research but not yet described in published literature, software must be made available to editors and reviewers. We strongly encourage code deposition in a community repository (e.g. GitHub). See the Nature Portfolio guidelines for submitting code & software for further information.

Data

Policy information about availability of data

All manuscripts must include a data availability statement. This statement should provide the following information, where applicable:

- Accession codes, unique identifiers, or web links for publicly available datasets
- A description of any restrictions on data availability
- For clinical datasets or third party data, please ensure that the statement adheres to our policy

The source data for all relevant statistics and example recordings are provided in the Source Data file, and are also available at the Gihub repository: https://github.com/HanLabBU/Shroff-Lowet-Nature-Communication-2023. The experimental raw data that support the findings of this study are available from the lead contact upon request.

Human research participants

Policy information about studies involving human research participants and Sex and Gender in Research.

Reporting on sex and gender	N/A
Population characteristics	N/A
Recruitment	N/A
Ethics oversight	N/A

Note that full information on the approval of the study protocol must also be provided in the manuscript.

Field-specific reporting

Please select the one below that is the best fit for your research. If you are not sure, read the appropriate sections before making your selection.

☒ Life sciences ☐ Behavioural & social sciences ☐ Ecological, evolutionary & environmental sciences

For a reference copy of the document with all sections, see nature.com/documents/nr-reporting-summary-flat.pdf

Life sciences study design

All studies must disclose on these points even when the disclosure is negative.

Sample size	Sample size in terms of neurons and subjects (mice) are declared in the manuscript. No power analysis for the number of mice was performed, and the number of mice used was determined by the convention of the field. The number of neurons analyzed was sufficient to identify differences between experimental conditions as supported by the statistical tests performed.
Data exclusions	Voltage imaging datasets with significant image motion or with low spike-to-baseline ratio were excluded from analysis. Detailed exclusion criteria were stated in the manuscript.
Replication	All attempts at replication were successful as described in the details of the statistical tests provided.
Randomization	Experimental data collection across days were randomized.
Blinding	Same analysis was performed across experimental conditions. Blinding is not relevant for this study.

Reporting for specific materials, systems and methods

We require information from authors about some types of materials, experimental systems and methods used in many studies. Here, indicate whether each material, system or method listed is relevant to your study. If you are not sure if a list item applies to your research, read the appropriate section before selecting a response.

Materials & experimental systems

n/a	Involved in the study	
☐	☒	Antibodies
☒	☐	Eukaryotic cell lines
☒	☐	Palaeontology and archaeology
☐	☒	Animals and other organisms
☒	☐	Clinical data
☒	☐	Dual use research of concern

Methods

n/a	Involved in the study	
☒	☐	ChIP-seq
☒	☐	Flow cytometry
☒	☐	MRI-based neuroimaging

Antibodies

Antibodies used	Staining of SPNs was performed with primary antibody rabbit anti-DARPP-32 (1:500, Abcam ab40801 [EP720Y]), followed by the secondary antibody AlexaFluor568 (1:500, goat anti-rabbit IgG, Invitrogen A11011).

Validation	All antibodies were used according to the protocols that have been validated by suppliers. The primary antibody rabbit anti-DARPP-32 (per the supplier website) was validated in mouse, rat, and human. The secondary antibody AlexaFluor568 (per the supplier website) is validated to react specifically with rabbit.

Animals and other research organisms

Policy information about studies involving animals; ARRIVE guidelines recommended for reporting animal research, and Sex and Gender in Research

Laboratory animals	Adult male and female C57BL/6 mice (Charles River Laboratories, Inc.), ChAT-Cre mice (ChAT-cre; Tg(Chat-cre)GM24Gsat/Mmucd, MMRRC_017269-UCD, NIH MMRRC), ChAT-tdT mice (crossed between ChAT-Cre and the tdT mouse line: B6.Cg-Gt(ROSA)26Sor-tm14 (CAG-tdTomato)Hze- /J from The Jackson Laboratory). Mice were 10-22 weeks at the start of the study.
Wild animals	No wild animals were used in this study
Reporting on sex	Experiments were performed on both male and female mice and sex was not a factor of the study.
Field-collected samples	No field samples were used in this study
Ethics oversight	All animal experiments were approved by the Boston University Institutional Animal Care and Use and Biosafety Committees

Note that full information on the approval of the study protocol must also be provided in the manuscript.
